# Optimization of bandgap reduction in 2-dimensional GO nanosheets and nanocomposites of GO/iron-oxide for electronic device applications

**DOI:** 10.1038/s41598-023-33200-4

**Published:** 2023-04-28

**Authors:** Sana Zainab, Muhammad Azeem, Saif Ullah Awan, Syed Rizwan, Naseem Iqbal, Jamshaid Rashid

**Affiliations:** 1 Department of Electrical Engineering, College of Electrical and Mechanical Engineering, National University of Sciences and Technology (NUST), Islamabad, 44000 Pakistan; 2 Department of Physics, School of Natural Sciences (SNS), National University of Sciences and Technology (NUST), Islamabad, 44000 Pakistan; 3 US-Pakistan Centre for Advanced Studies in Energy (USPCAS-E), National University of Sciences and Technology (NUST), Islamabad, 44000 Pakistan; 4 Department of Environmental Sciences, Faculty of Biological Sciences, Quaid-i-Azam University, Islamabad, 45320 Pakistan; 5 BNU-HKUST Laboratory for Green Innovation, Advanced Institute of Natural Sciences, Beijing Normal University at Zhuhai, Zhuhai, 519087 China

**Keywords:** Energy science and technology, Engineering, Materials science, Nanoscience and technology

## Abstract

In this report we have developed different fabrication parameters to tailor the optical bandgap of graphene oxide (GO) nanosheets to make it operational candidate in electronic industry. Here we performed two ways to reduce the bandgap of GO nanosheets. First, we have optimized the oxidation level of GO by reducing amount of oxidizing agent (i.e. KMnO_4_) to control the sp^2^/sp^3^ hybridization ratio for a series of GO nanosheets samples. We noticed the reduction in primary band edge 3.93–3.2 eV while secondary band edge 2.98–2.2 eV of GO nanosheets as the amount of KMnO_4_ is decreased from 100 to 30%. Second, we have fabricated a series of 2-dimensional nanocomposites sample containing GO/Iron-oxide by using a novel synthesis process wet impregnation method. XRD analysis of synthesized nanocomposites confirmed the presence of both phases,$\alpha$-Fe_2_O_3_ and Fe_3_O_4_ of iron-oxide with prominent plane (001) of GO. Morphological investigation rules out all the possibilities of agglomerations of iron oxide nanoparticles and coagulation of GO nanosheets. Elemental mapping endorsed the homogeneous distribution of iron oxide nanoparticles throughout the GO nanosheets. Raman spectroscopy confirmed the fairly constant I_D_/I_G_ ratio and FWHM of D and G peaks, thus proving the fact that the synthesis process of nanocomposites has no effect on the degree of oxidation of GO flakes. Red shift in G peak position of all the nanocomposites samples showed the electronic interaction among the constituents of the nanocomposite. Linear decrease in the intensity of PL (Photoluminescence) spectra with the increasing of Iron oxide nanoparticles points towards the increased interaction among the iron oxide nanoparticles and GO flakes. Optical absorption spectroscopy reveals the linear decrease in primary edge of bandgap from 2.8 to 0.99 eV while secondary edge decrease 3.93–2.2 eV as the loading of $\alpha$-Fe_2_O_3_ nanoparticles is increased from 0 to 5% in GO nanosheets. Among these nanocomposites samples 5%-iron-oxide/95%-GO nanosheet sample may be a good contestant for electronic devices.

## Introduction

Graphene in its monolayer form is a zero bandgap material with *sp*^2^–*sp*^2^ bonding between carbon atoms. For its use in semiconductor devices, its bandgap has to be opened by functionalization of oxygen in formation of Graphene oxide (GO). This oxygen functionalization results in *sp*^*3*^–*sp*^3^ bonding between atoms and almost insulating properties with a very high bandgap. To reduce this bandgap, it is most important to control the amount of oxygen/carbon (O/C) ratio without reducing GO into reduced GO (known as r-GO). A linear relationship between oxygen concentration and bandgap of GO is observed theoretically with linear increase in bandgap with increase in oxygen to carbon ratio^1,2^. This increase in bandgap is observed due to localization of electronic states and weak bonding between C–C atoms. This weak bonding results from interaction between π orbital of graphene and 2p_z_ orbital of Oxygen from epoxy group. With increase in O/C ratio up to or more than 50% in GO, bandgap also transition from direct to indirect^3,4^. Stacking configuration of GO layers also have effect on bandgap with AA stacking being more suitable than AB due to loosening of unoccupied stated near fermi level^5^. Its low cost and large scale production method make it favorable for electronic devices applications. But the optical bandgap of GO is far greater than it is required to operate as a semiconductor in electronic devices.

Fabrication of GO aerogels with ordered structures (e.g. radial and centrosymmetric) using freeze casting method had been demonstrated by Wang et al.^6,7^. Recent developments in 3D printing of graphene and its derivatives based materials and possible potential of their applications for batteries, supercapacitors, solar steam generators, and electro-thermal conversion are comprehensive reviewed^8^. Using rapid heating technique Chen et al.^9^ had produced uniform, expanded, and reduced GO films of a desired thickness to a given height by the use of a physical barrier, followed by compression to create a dense paper-like material with a low oxygen content and a greater content of carbon sp^2^ hybridization that provides a route for fabricating “graphenic” foils of different thicknesses that are likely to be useful for many applications.

Usually, nanocomposites provide extra ordinary results as they have improved structural, optical, electrical, thermal, photocatalytic and mechanical properties. Nanocomposites of nickel–iron sulfide and carbon nanotubes are revealed for application of supercapacitors^10^. High atomic level Fe (Iron) loading density for the understanding of electronic structure modulation and intrinsic activity aimed at high-efficiency catalyst are designed toward oxidation-related reactions^11^. Pen et al.^12^ had developed a novel spin regularization strategy to prepare regularized GO membranes with ordered and stable laminar structures through uniform interlayer mass transfer channels (exhibits a high gravimetric capacitance) for high-performance electrodes for obtaining high electron and charge transfer capability that can meet the increasing requirement for high-rate energy storage devices. Zhang et al.^13^ proposed 3D interconnected N, S co-doped branched carbon nanotubes structure with uniformly distributed active sites can form intimate contact interface and interconnected channels, thus facilitate the electron transfer and electrolyte infiltration for applications of energy devices. Lv et al.^14^ had fabricated GO membranes by insertion of various metals (e.g. Cu, Fe, Ni, and Zn) and obtained improved high aqueous stability and high separation performance due to cation-π coordination and electrostatic interaction between metal cations and GO nanosheets.

Bandgap of 2D materials can be tuned by doping, surface modification, defects engineering and nanocomposite formation. Bandgap of MoS_2_ was reduced by surface doping of oxygen atoms. Surface doped oxygen atom enhanced the intrinsic conductivity of MoS_2_ confirming the significant effect of surface doping on the electronic structure^15^. Another way of reducing bandgap of nanomaterials is by incorporating material having narrow bandgap. The introduction of narrow bandgap material generates impurity energy levels above the valence band edge, resulting in less energy required to excite charge carriers hence decreasing optical bandgap^16^. Many nanomaterials are being employed to reduce the bandgap of other materials. Graphene is being used to reduce optical bandgap of TiO_2_ for enhanced photocatalytic activities. 2D/0D type hetero-structures are also in use to tailor optical bandgap of materials. Adding r-GO in wide bandgap of g-C_3_N_4_ nano-dots resulted in narrowing of bandgap of nanocomposite^17^. In similar trend, optical band-gap of the r-GO/TiO_2_ film decreases linearly with increasing r-GO proportion. Here r-GO acts as narrow bandgap material and increasing its value results in increase and shift in density of State (DOS) distribution towards the gap, resulting in the conduction band (CB) edge to move to a significant level of a new mobility edge and restore the initial electron effective DOS^18^.

Here are many other techniques applied to reduce the bandgap of GO. Hasan et al.^19^ propose that ozone-induced functionalization decreases the size of graphitic islands affecting the GO band gap and emission energies, based on this model of GO fluorescence originating from *sp*^2^ graphitic islands confined by oxygenated addends. The optical band gap of GO can be reduced and tuned effectively from 2.7 to 1.15 eV, by its reduction with mild reagents e.g. glucose, fructose and ascorbic acid while NH_4_OH speeds up the reaction^20^. The chemical bond C-Ti between the titanium oxide and graphene sheets is at the origin of bandgap reduction of TiO_2_ Assembled with Graphene Oxide Nanosheets^21^.Auspiciously, Iron(III) chloride is photochemically active and have quantum yields for FeCl^+^ are 0.048 at 300 nm and 0.038 at 340 nm. The quantum yields for FeCl^2+^ are 0.088 at 300 nm and 0.051 at 340 nm. The presence of FeCl^2+^ and Fe(Cl_2_)^+^ species will result in an enhanced photoreduction rates due to a shift in action spectra to longer wavelengths around 340 nm. To some extent, graphene and its derivatives can also be considered as super-aromatic molecules, and have potential to react with the Fenton reagent^22^. Recently, the Fenton reaction has been successfully employed to investigate the effect of hydroxyl radical (HO•) on the surface structure of multi-walled carbon nanotubes (MWNTs) and fabricate graphene quantum dots (GQDs)^22^.Moreover, compared with the classical Fenton method, theIron(III) chloride like other + 3 oxidation state like other systems ferrioxalate-mediated photo-Fenton method exhibits much higher etching efficiency due to more efficient and stable HO• production benefiting from the highly efficient regeneration of ferrous ions. To be specific, the classical Fenton (the ferrous and/or ferric cation decomposes catalytically hydrogen peroxide to generate powerful oxidizing agents) method produces HO• mainly via the Haber–Weiss reaction. Meanwhile, Fe(II) can be regenerated from Fe(III) through the Fenton-like reaction^22^.

In GO based composites physical properties could be improved more even for very small amount of loading of the filler. In this article we have used narrow bandgap property of Iron oxide nanoparticles and used it to further reduce the bandgap of GO without reduction.In this article,we have designed and performed reproducible scheme to tailor the optical bandgap of GO to make it usable in electronic industry. This composite formation technique is designed by keeping one thing in mind, to make GO nanocomposite without its reduction. In this method suspension of GO and Iron oxide are mixed together using ultra sonication. Nanocomposites formed by this technique have reduced band gap owing to the electron transfer among the heterojunction interfaces while retaining the *sp*^3^ hybridization of GO. Both series of samples are characterized for structural, morphology, compositional analysis and optical properties. Optical bandgap of GO is highly dependent on the *sp*^2^/*sp*^3^ hybridization ratio of carbon atoms. Theoretically if we could somehow control the degree of oxidation during the synthesis of GO its optical bandgap could be controlled. This reduction of optical band-gap can be credited to electronic interactions between the composite materials during synthesis process.

## Results and discussion

### X-ray diffraction analysis

**Figure 1:**
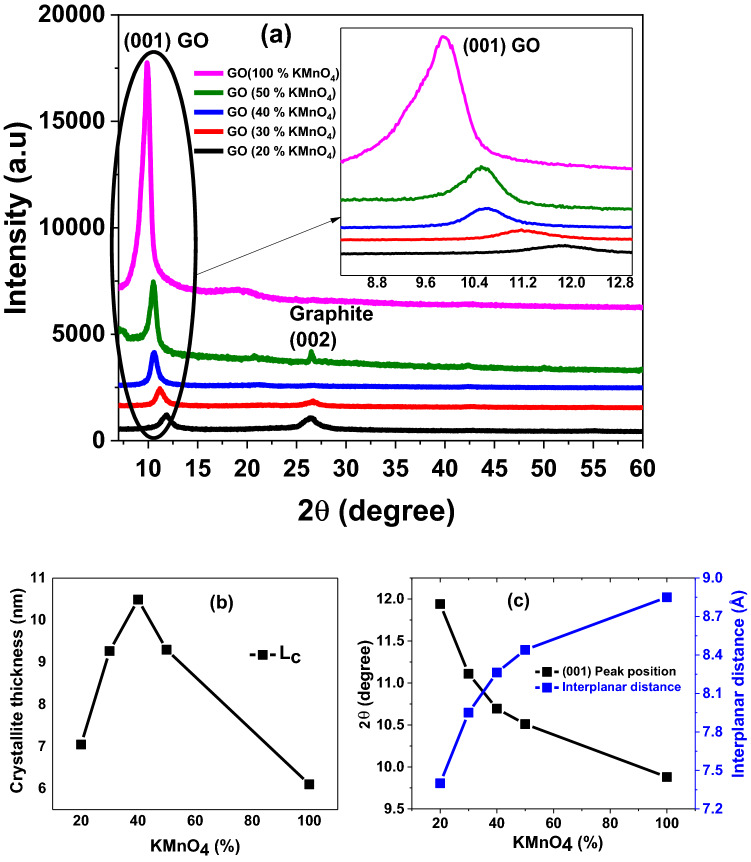
(**a**) XRD spectra of a series of controlled oxidized GO nanosheets samples with varying KMNO4 concentration (**b**) Crystallite height (**c**) Peak position of (001) plane and interplanar distances of controlled oxidized GO nanosheets samples.

To analyze the phase formation of GO nanosheets and interplanar distance for our samples, we performed X-ray Diffraction (XRD) measurements. Figure 1a shows the XRD crystal spectra of as synthesized GO samples. We observed that most prominent plane (001) correspond to GO with another minor peak related to (002) plane of graphite as reported earlier^23^. It is also noted that the peak intensity peak of GO (001) plane increases as we increase the concentration of KMnO_4_ from 20 to 100% while overall the opposite trend of graphitic (002) plane is observed. This observation clearly indicates that at higher concentration of KMnO_4_ more single prominent GO phase formation is stabilized. The variation in peak positions of GO (001) plane is more elaborated as revealed inset of Fig. 1a, may be owing to the different degree of oxidation. It is observed that (001) plane shifts toward higher 2$\uptheta$ as the amount of oxidant is decreased. Ayrat group^24^ explained these two phases and reported that oxidation of graphite flake starts from the edge and moves toward the center. As the amount of oxidization is low only edges of graphite flakes gets oxidized and center portion remains un-oxidized. This uneven oxidation is not carried out for all the flakes, some flakes get fully oxidized while other remains partially oxidized. These partially oxidized flakes have un-oxidized *sp*^2^ hybridized graphitic domain in them surrounded by *sp*^3^ hybridized GO. This results in the presence of both GO and graphite in the diffraction spectra of the samples.

The crystallite thickness is measured and demonstrated in Fig. 1b. We observed that crystallite thickness is highest (~ 10.5 nm) for 40%- KMnO_4_ and smallest (~ 6 nm) for 100%- KMnO_4_ concentrations. Peak position of (001) plane and interplanar distance of controlled oxidized GO nanosheets samples are showing opposite behavior with increasing %ages of KMnO_4_ as presented in Fig. 1c. As the amount of KMnO_4_ is increased may be greater functional groups gets attached to the basal plane thus enlarged the interplanar distance as stated by Marcano et al.^23^. Here we may argue the increase in *d*-spacing with an increase in the amount of oxidizing agent. In other words we can say that with the increase in oxidizing agent, *d*-spacing increases so possibly *sp*^2^ to *sp*^3^ hybridization ratio may decrease which may be responsible for increasing the optical bandgap of these nanosheets GO samples.

Figure 1Sa represents the XRD spectra of analytical grade Graphite powder of Sigma-Aldrich. This XRD spectra shows three significant peaks at 26.38$^\circ$, 44.25$^\circ$ and at 54.54$^\circ$ identified as (002), (101) and (004) planes of graphite respectively according to the JCPDS card no 01-075-1621. To get information regarding oxidation level and the effects of chemical processes performed throughout the synthesis of GO and its nanocomposites, determination of interplanar distance (d) is of great importance, so Graphite sample is used as a reference for that purpose. For the determination of interplanar distance Bragg’s law

1 $$(n\lambda =2d\mathrm{sin}\theta)$$

is used^25^. Here $\theta$ is the one half of peak diffraction angle, d is the interplanar distance, $\lambda$ represents the Cu K(α) wavelength, which in our case is 1.54 Å and n is the mode of vibration. For all the analysis second mode of vibration is used. The interplanar distance calculated is 0.337 nm for (002) plane and 0.358 nm for (004) plane, these values corresponds to the JCPDS card no 01-075-1621. In order to calculate the number of layer in the crystallite, L_c_ lattice parameter in z-axis or the crystallite height is required. Scherer’s equation

2 $$L_{C} = 0.89\mathchar'26\mkern-10mu\lambda /\beta \cos \theta$$

can be used to calculate the crystallite height for graphitic materials because of their planner structure^25–30^. In this relation 0.89 is the Scherrer’s constant used for L_c_ of graphitic materials,$\lambda$ is the wavelength of Cu K(α), β is the graphically calculated full width and half maxima of the peak. Average crystallite height is found ~ 26.78 nm for pure graphite. To find out the number of layers lattice spacing “d” and crystallite height “L_c_” is used in the following relation

3 $$N=\frac{Lc}{D}+1$$

According to this relation, there are around 151 layers in an average graphite crystallite. Figure 1Sb shows the XRD spectra of as synthesized GO flakes carried out at 2$\theta$ value of 5$^\circ$ to 70$^\circ$. A single prominent peak at 9.88° is identified to be as (001) plane of GO. Peak position and intensity corresponds to the earlier reported values^23^. Inter-planner distance calculated with the help of Bragg’s law showed a substantial increase in “d” spacing from 0.33 nm of Graphite to 0.885 nm of GO. This rise in inter planar distance is because of the hydroxyl and epoxy functional groups attached on the basil plane of honey comb lattice of graphite during its chemical exfoliation^23^. These functional groups make bonds with the Π electrons of the carbon atoms. This results in the change in hybridization of carbon atoms from *sp*^2^ to *sp*^3^. This change of hybridization alters the band gap of the material, zero band gap Graphite becomes semiconductor with a band gap ranging to about 3.7 eV. Degree of oxidation or the amount of functional groups attached to the basal plane of GO could be easily monitored with the help of inter planner distance. Higher oxidation levels leads toward the increased d-spacing and vice versa. This d-spacing also identifies any kind of reduction in GO in reduction process functional groups gets removed from the basal plane of GO thus in turn causing the interplanar distance to decrease. Scherrer’s formula gives the approximated L_c_ value of 6.099 nm for as-synthesized GO nanosheets. Here we can see a substantial decrease in crystallite thickness as compared to its precursor. This decreased crystallite thickness is an indicator for a successful chemical exfoliation. This exfoliation is carried by the exothermic oxidation reaction. We found that instead of 151 layers in untreated commercial grade Graphite; GO only have about 7 layers of *sp*^3^ hybridized carbon atoms. The XRD spectra of as synthesized iron oxide nanoparticles obtained as given in Fig. 1Sc. After very careful analysis it is found that as synthesized iron oxide nanoparticles contained α-Fe_2_O_3_ and Fe_3_O_4_ phases by comparing with JCPDS card no-65-3107 and 89-0598. Both phases of iron oxide in pure nanoparticles sample have been marked in Fig. 1Sc. Presence of Fe_3_O_4_ in the specimen is because of the further oxidation of α-Fe_2_O_3_ during the synthesis process. Crystallite size for these Iron oxide nanoparticles is found with the help of Scherrer’s equation. Analysis reveals the presence of nanoparticles in the specimen with an average size of 28.1 nm.

**Figure 2:**
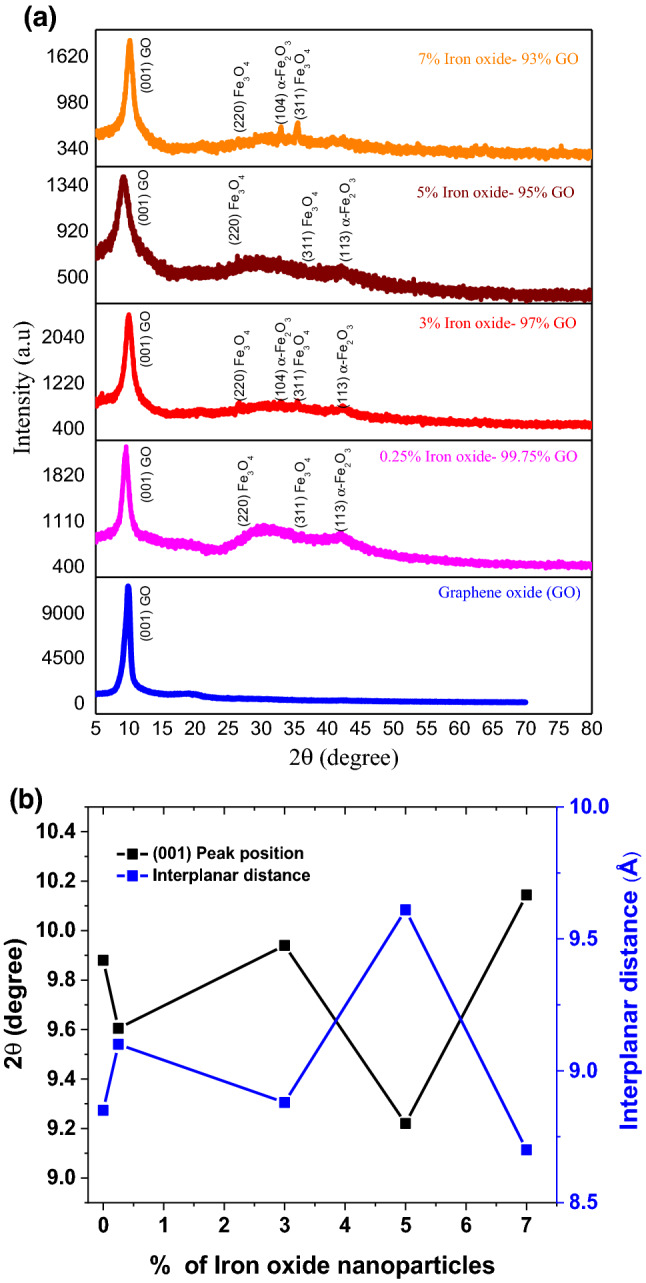
(**a**) XRD spectra of nanocomposite (GO/Iron-oxide) (**b**) Interplanar distance between sheets of graphene oxide in nanocomposite samples.

XRD spectra of GO/iron-oxide nanocomposites synthesized by using “wet impregnation technique” are shown in Fig. 2a for various compositions of 0.25, 3, 5 and 7% Iron-oxide concentration. Presence of (001) characteristic peak of GO at 9.6$^\circ$ indicates the fact that no reduction of GO (i.e. r-GO) is carried out during the nanocomposite synthesis process^23^. Peaks at 30.3$^\circ$, 36.3$^\circ$ and 39.63$^\circ$ turns out to be (220) and (311) of Fe_3_O_4_ and (113) planes of α-Fe_2_O_3_ respectively, when compared with JCPDS card no-65-3107 and 89-0598. Interplanar distance of the nanocomposite calculated is 0.91 nm as compared to 0.885 nm of pure GO. This slight increase is because of the penetration of iron oxide nanoparticles between the layers of GO. Crystallite thickness of the nanocomposite is 6.95 nm which is also slightly greater than the pure GO sample. For 3% α-Fe_2_O_3_ and 97% GO nanocomposite synthesized, Peaks at 33.1°, 35.5° and 42.3° corresponds to (104), (110) and (202) peaks of α-Fe_2_O_3_ when compared with JCPDS card no-65-3107. Interplanar distance calculated with the help of Bragg’s law is 0.88 nm and L_c_ value is 5.37 nm. These values are similar to that of pure GO. These values also suggests that any kind of structural changes have not occurred during the nanocomposite formation and iron oxide nanoparticles are present only on the surface of GO. The diffracted peaks for nanocomposite samples 5 and 7% Iron-oxide concentration has been marked in Fig. 2a. Interplanar distance and crystallite height for 5% iron oxide-95% GO sample is 0.96 and 4.64 nm respectively and for 93% GO%-7% iron oxide nanocomposite d-spacing is 0.84 nm and L_c_ parameter is 3.34 nm. The data of prominent peak position of GO and interplanar distances for variations compositions of nanocomposites extracted from Fig. 2a has been elaborated in Fig. 2b. From Fig. 2, we may infer that 5% nanocomposite sample has best options among series of samples, where we noticed highest *d*-spacing.

### Micro-structural properties

**Figure 3:**
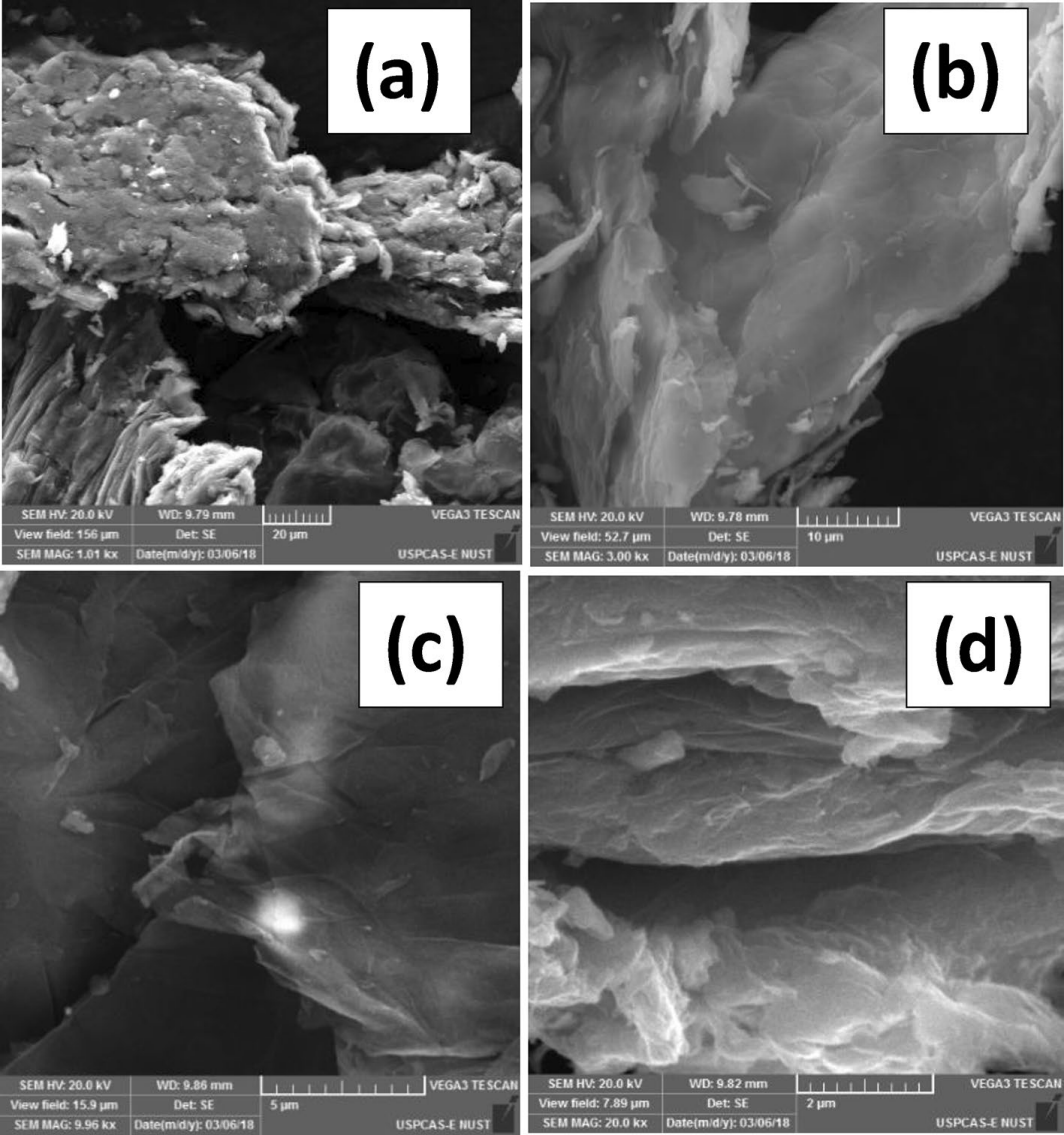
SEM images of pure graphene oxide nanosheets at different scale (**a**) 20 μm (**b**) 10 μm (**c**) 5 μm (**d**) 2 μm.

**Figure 4:**
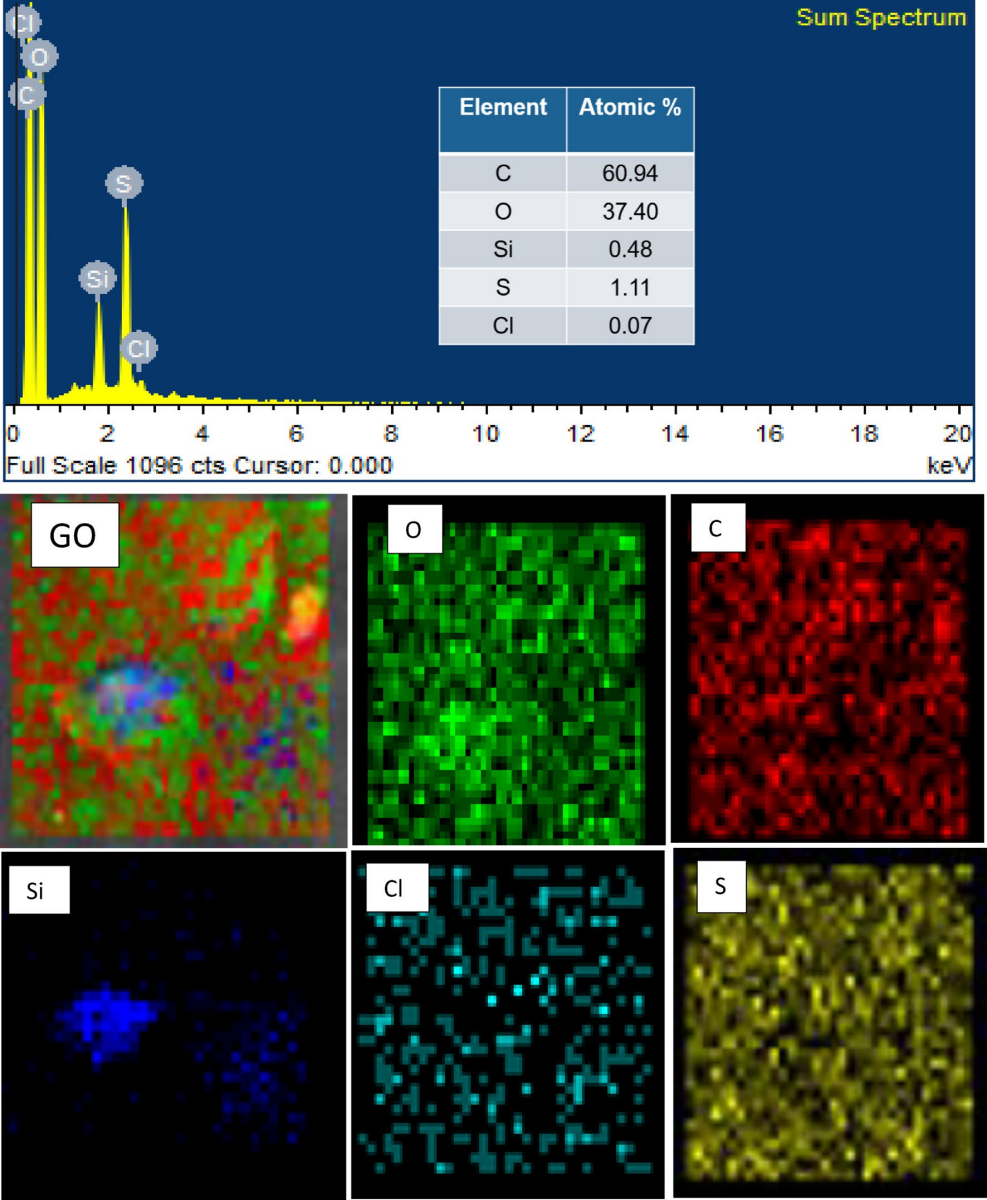
EDX spectra of pure graphene oxide nanosheets. Elemental mapping of pure GO sample reflects the presence of different constituents in GO as O (oxygen), C (Carbon), Si (Silicon), Cl (Chlorine) and S (Sulfur).

To observe the morphology, the elemental distribution of nanocomposites and regional reduction of graphene oxide flakes during nanocomposite synthesis Field Emission Scanning Electron Microscopy (FE-SEM) and energy dispersive spectroscopy (EDS) measurements were performed for entire series of GO nanosheets samples and GO/Iron-oxide nanocomposites samples. Figure 3a–d represents the SEM images of GO nanosheets at different scale (a) 20-μm (b)10-μm (c) 5-μm (d) 2-μm. We observed that nanosheets are not fully separated from each other but still agglomeration was present at small scale. These GO flakes were further exposed to EDS to know the elemental composition. This study provides valuable information about the elemental composition of GO. The EDS spectra and elemental mapping of GO nanosheets had been demonstrated in Fig. 4. Here we noted that GO nanosheets sample showed the presence of higher amount of carbon and oxygen. Presence of Sulfur and chlorine is because of Sulfuric acid and hydrochloric acid used in the synthesis scheme whereas silicon is because of the impurities present in the precursor graphite^31^. Hydrogen present in hydroxyl functional groups attached on the basal plane of GO flakes is undetectable because of its lower atomic mass. The percentage presence of normal concentration and atomic concentration is given accordingly in table inset EDS spectra (Fig. 4).

**Figure 5:**
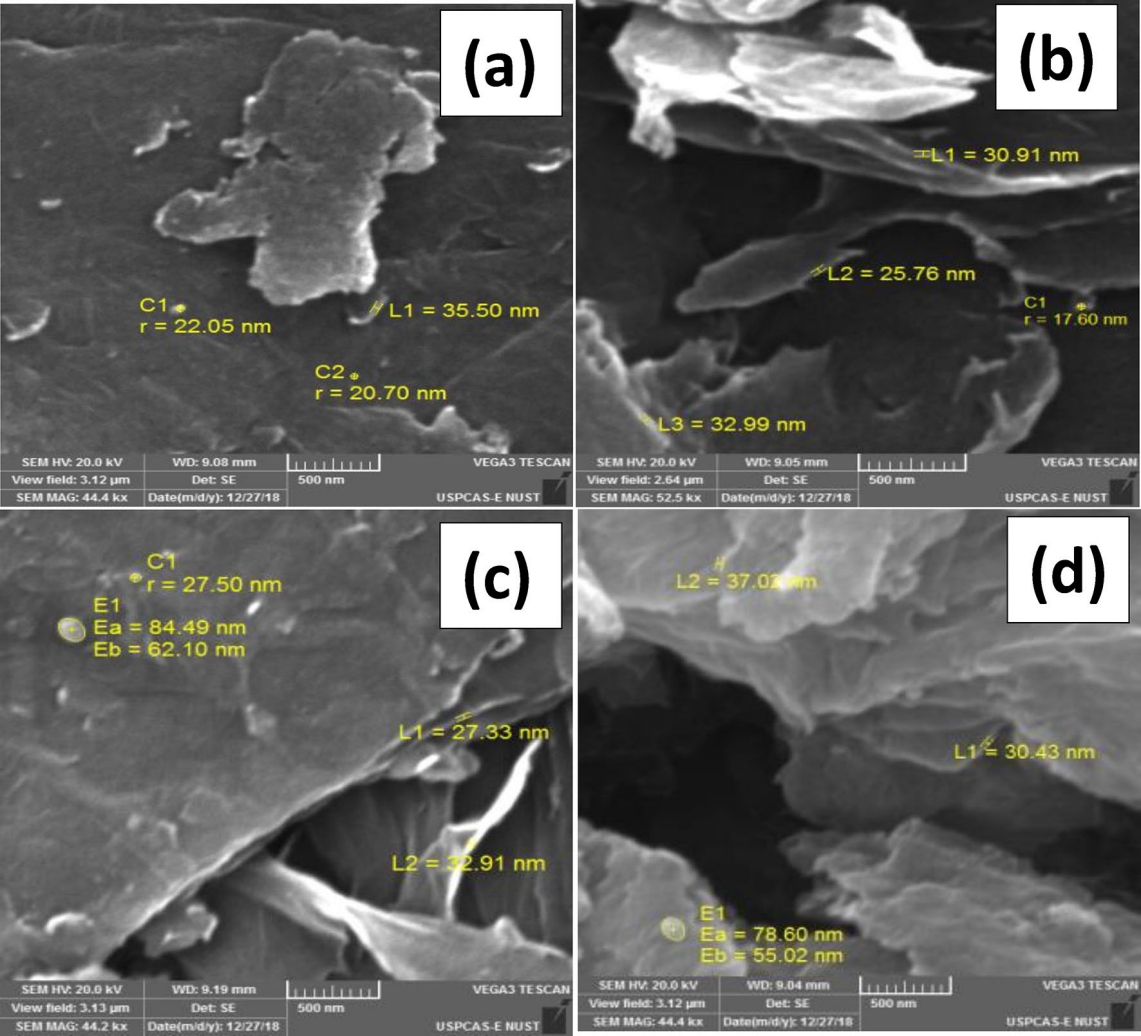
SEM images of GO/Iron Oxide nanocomposites samples for various concentration of iron oxides (**a**) 0.25% (**b**) 3% (**c**) 5% (**d**) 7%.

**Figure 6:**
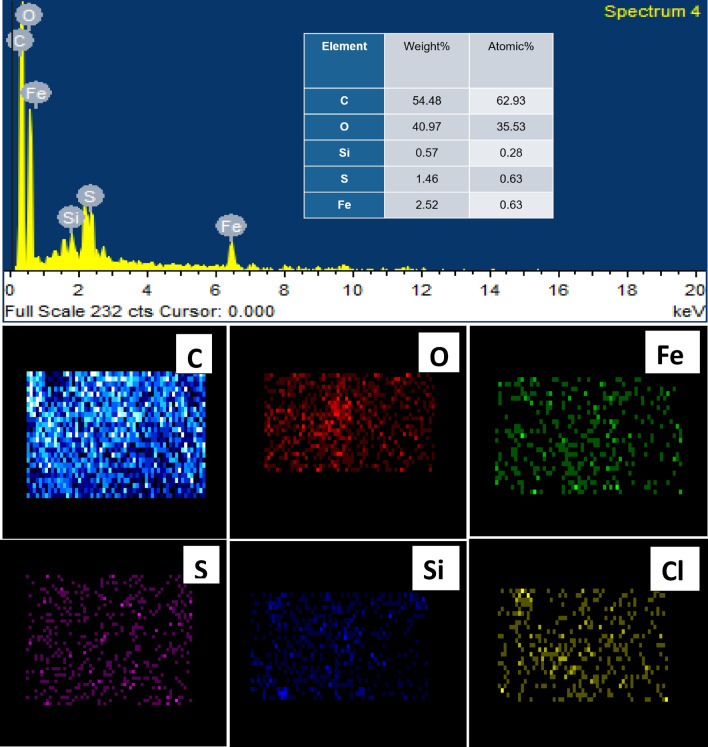
EDX spectra of composite of GO/Iron-oxide nanocomposites. Elemental mapping of reflects the presence of different constituents, C (Carbon), O (oxygen), Fe (Iron) S (Sulfur) Si (Silicon) and Cl (Chlorine) in nanocomposite sample.

Figure 5a–d is the SEM images of GO/Iron-oxide nanocomposites at 500 nm scale for various concentrations of iron oxides (a) 0.25% (b) 3% (c) 5% (d) 7%. We noted the agglomeration among nanosheets. GO flakes are evenly decorated with iron oxide nanoparticles. More concentration of nanoparticles of iron oxide is visible in Fig. 5d i.e. 93%GO/7%-Iron-oxide nanocomposites as compared to Fig. 5a i.e. 99.75%GO/0.25%-Iron-oxide nanocomposites sample. Any sign of reduction is not present in any of the sample, thus proving the results of XRD. GO flakes are fully oxidized and have around 25–32 nm thickness. Particle size of iron oxide nanoparticles and sheet thickness of GO flakes observed with the help of SEM images are in accordance to the XRD results. The EDS spectrum and elemental analysis of 95%GO/5%-Iron-oxide nanocomposites sample had been presented in Fig. 6. The EDS spectrum represents very prominent peaks of carbon, oxygen and iron. Generic impurities of GO, silicon and sulfur are also present in the spectrum^31^. Elemental mapping and atomic percentages of these constituent elements are demonstrated in Fig. 6. Even distributions of oxygen atoms throughout the flake rules out any chance of regional reduction of GO caused by the synthesis process or due to the oxidation of α-Fe_2_O_3_. Atomic percentages of these constituent elements are shown in table inset EDS spectra (Fig. 6). Higher carbon to oxygen ratio proves the XRD results that no reduction of GO is carried out during the nanocomposite formation. Sulfur is because of sulfuric acid used in the synthesis of GO and silicon is present in the nanocomposites because silicon based compounds are found in the graphite used as a starting material for GO synthesis.

### Raman spectroscopy

**Figure 7:**
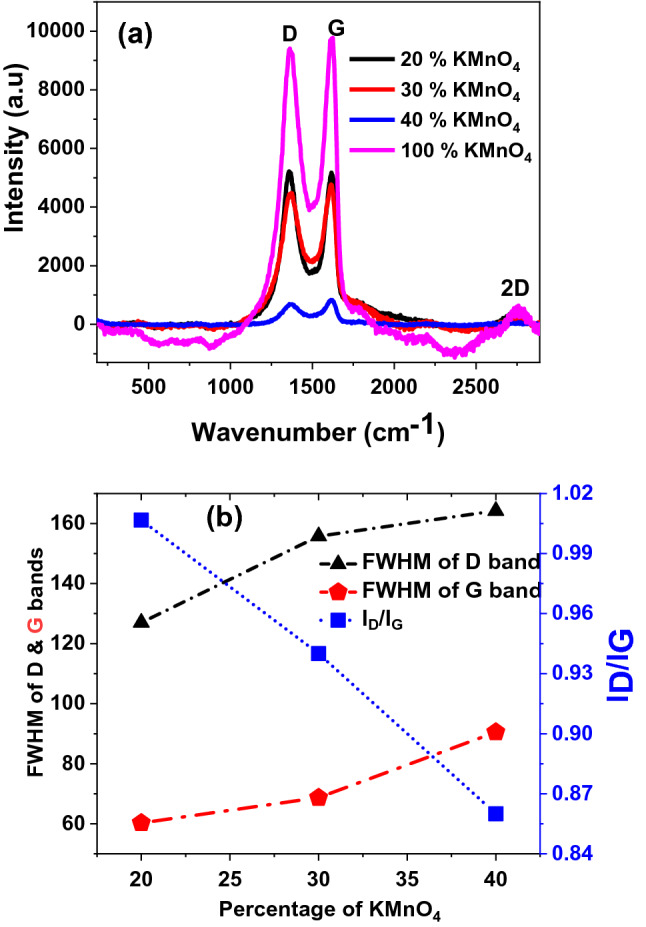
(**a**) Room temperature Raman spectra of a series (varying the % ages of KMNO_4_) of controlled oxidized GO nanosheets samples (**b**) Variation in FWHM of D and G bands and I_D_/I_G_ ratio of controlled oxidized GO nanosheets samples versus concentration of KMNO_4_.

To identify the degree of oxidation and to observe the un-oxidized domain size variation during the controlled oxidation of GO nanosheets samples and GO/Iron-oxide nanocomposites samples, Raman spectroscopic measurements were accomplished at room temperature with LASER excitation wavelength of 514 nm. Raman spectra for various samples of GO nanosheets with varying KMnO_4_ concentrations for entire range 400–3000 cm^−1^ had been exhibited in Fig. 7a. The spectrum of each sample reveals the presence of D, G and 2D characteristics peaks of GO nanosheets as reported previously^9–19^. The peak at 1366 cm^−1^ position corresponds to D-band that may be assigned by A_1g_ mode of vibrations. The formation of D-band is probably due to the defect generated phonon mode vibrations or disorderness in the honeycomb graphitic structures such as vacancies, bond-angle disorder, edge defects and bond-length disorder^32–35^. Another reason of D-band can be the presence of hydroxyl and epoxide functional groups on the basal plane of GO nanosheets^36^. Another most prominent peak at 1617 cm^−1^ position linked with G-band that may be caused by the first order C–C stretching vibrations of E_2g_ phonons observed for *sp*^2^ carbon domains^35,37–39^. Moreover, another 2D peak is observed at 2755 cm^−1^ and it is assigned to second-order zone boundary phonons or to a two phonon double resonance process^40^.

Both the Full width half maximum (FWHM) of D-band and G-band, intensity ratios of D-band to G-band (I_D_/I_G_) versus KMnO_4_ concentrations have been plotted in Fig. 7b. From this analysis we noted that FWHM values of both bands increases as % age of KMnO_4_ increases. On getting this information we may propose that degree of oxidation may increase as FWHM of D and G bands increase. In previous studies, FWHM of D-band of GO is directly related to the degree of oxidation because the oxidation process has a direct effect on *sp*^2^/*sp*^3^ ratio^41^ i.e. the reduction of *sp*^2^/*sp*^3^ hybridization ratio of GO flakes. Similarly, the FWHM of G-band also increased with increasing the degree of oxidation level of GO nanosheets^42^. We found the I_D_/I_G_ ratio reflected opposite trend as that of FWHM of bands with the increase of % ages of KMnO_4_. The I_D_/I_G_ ratio of GO nanosheets is also associated with the degree of oxidation and in-plane *sp*^2^ domain size^38,41^. As the degree of oxidation increases I_D_/I_G_ ratio decreases representing the decrease in in-plane *sp*^2^ domain size. On the basis of above Raman results we may argue that degree of oxidation in GO samples increased as we increased the KMnO_4_ concentrations in samples.

**Figure 8:**
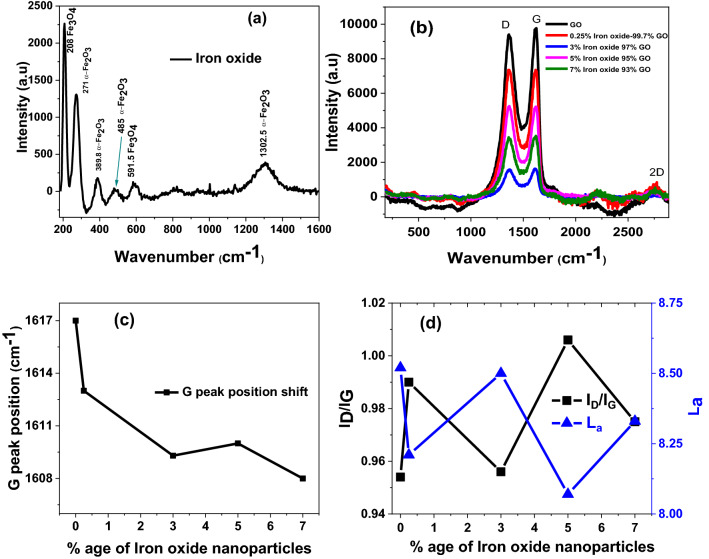
Raman spectra of (**a**) Iron oxide (**b**) nanocomposites samples (**c**) Raman peaks intensity ratio (**d**) G-peak position shift versus iron oxide concentration in nanocomposites series of samples.

Raman spectrum of Iron oxide nanoparticles is demonstrated in Fig. 8a. We analyzed the spectroscopic data and found that both phases of α-Fe_2_O_3_ and Fe_3_O_4_ are present as we extracted from XRD data. The characteristics peaks at positions of 271, 389.8, 485 and 1302 cm^−1^ represents the α-Fe_2_O_3_ phase while the peaks at 208 and 591.5 cm^−1^ belongs to Fe_3_O_4_ as reported in previous studies^43–45^. Peaks at 208 and 591 cm^−1^ corresponds to A_1g_ mode of vibrations while the remaining peaks except the one at 1302 cm^−1^ are assigned to E_g_ mode of vibrations and the peak at 1308 cm^−1^ corresponds to the two magnon scattering caused by the interaction of two magnons created on anti-parallel close spin sites inconsistent as reported in literature^46^. Figure 8b reflects the Raman spectra of GO and GO/Iron-oxide nanocomposites. Owing to the lower concentration of iron oxide nanoparticles, no characteristic peaks of iron oxide is visible in any of the Raman spectra of GO/Iron-oxide nanocomposites samples. Analysis to these spectra reveals the presence of D-band (1366 cm^−1^) is assigned to the A_1g_, G-band (1617 cm^−1^) is caused by the first order C–C stretching vibrations of E_2g_ phonons observed for *sp*^2^ carbon domains and 2D characteristics peaks (2755 cm^−1^) assigned to second-order zone boundary phonons or to a two phonon double resonance process of GO as reported in previously^32–40^. We measured the FWHM of D-peak is found to be 167 cm^−1^ and FWHM of G-peak is found to be 107 cm^−1^ for GO/Iron-oxide nanocomposites samples which correspond to reported values previously^32–40^.

G-band position of GO and GO/Iron-oxide nanocomposites had been plotted in Fig. 8c. Position of the G-band in Graphene based nanosheets systems is an ideal marker to study the interaction among nanomaterials. To check that whether our GO/Iron-oxide nanocomposites samples with proper electronic interaction is made or not, positions of G-band are compared. We found a red shift in the peak position of G-band as with increasing the Iron-oxide concentration in GO nanosheets structures. Similarly, electronic interaction between the carbon based materials and nanoparticles causes the G-band to red shift^47–53^. This red shift becomes greater as the interaction among the constituents of the nanocomposite increases a linear red shift in G-band position could be seen in Fig. 8c as the loading of iron oxide nanoparticles is increased from 0.25 to 7%. This analysis gives us information that iron oxide nanoparticles are not just resting on the basal plane of GO nanosheets but they are electronically interacting with each other.

Figure 8d showed the extracted data of intensity as well ratio of D and G peaks (I_D_/I_G_) ratio of GO/Iron-oxide nanocomposites samples while in general the I_D_/I_G_ ratio in GO nanosheets is inversely proportional to the degree of oxidation and in-plane *sp*^2^ domain size. As the degree of oxidation decreases I_D_/I_G_ ratio increases representing an increase in in-plane *sp*^2^ domain size^38,41^. This fairly similar I_D_/I_G_ ratio points towards the fact that no reduction of GO nanosheets is carried out during the synthesis process of nanocomposite nor by the chemical reaction between the $\mathrm{\alpha }-$Fe_2_O_3_ or Fe_3_O_4_ nanoparticles and GO as reported earlier^42,54,55^. The I_D_/I_G_ ratio could also be used for the determination of the size of *sp*^2^ domains in the GO based systems^41,56^. For this purpose many relations such as the Knight and White formula^57^ and Tuinstra and Koeng’s relation^39^ have been employed to measure the average *sp*^2^ domain size. Here in this work we have used a general relation, given by Cancodo et al.^58^ for the measurement of the average crystallite size of the *sp*^2^ domain. The expression for this relation is given below;

4 $$\rm L_{\rm a} (\rm nm)=[(2.4 ^{*} 10^{-10})(\lambda_{1})^4]/[\rm I_{\rm D}/ \rm I_{\rm G}]$$

Where L_a_ is the average crystallite size of the sp^2^ domains, $\uplambda$_l_ is the input laser energy, I_D_ is the intensity of the D-band, and I_G_ is the intensity of the G-band. In plane *sp*^2^ domain sizes of GO and GO/Iron-oxide nanocomposites samples calculated utilizing equation-5as are plotted in Fig. 8d. All the nanocomposites have L_a_ value fairly closer to the pristine GO nanosheets, thus suggests that no reduction of GO is carried out during the synthesis process of nanocomposites nor by the chemical reactions between the $\mathrm{\alpha }-$Fe_2_O_3_ or Fe_3_O_4_ nanoparticles and GO nanosheets^42,54,55^.

### Photoluminescence spectroscopy

**Figure 9:**
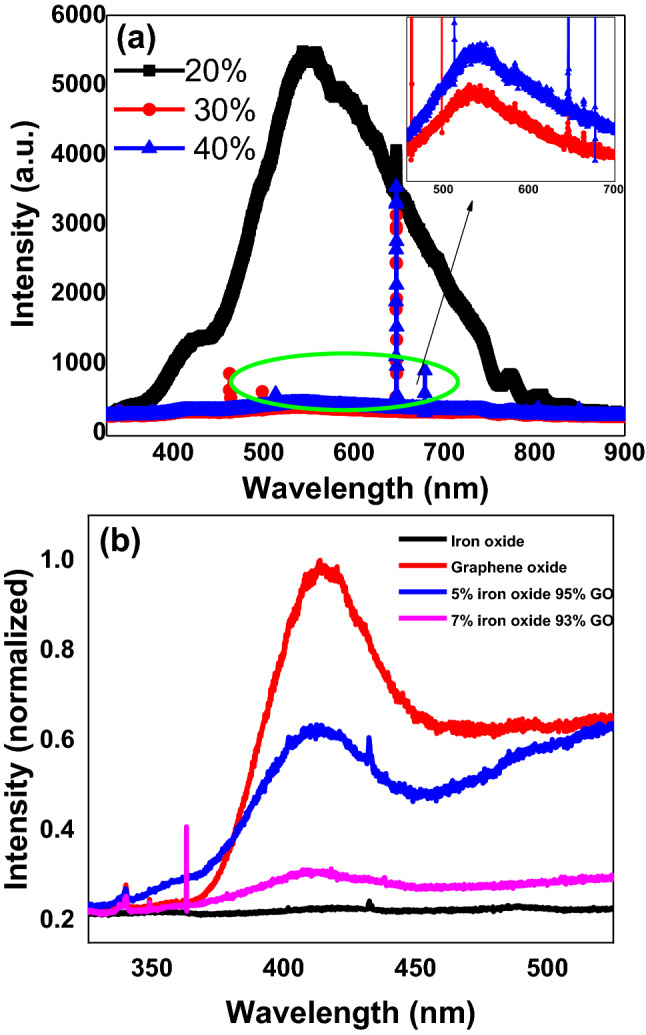
Photoluminescence (PL) spectra of (**a**) controlled oxidized GO nanosheets samples (**b**) Graphene oxide, Iron oxide nanoparticles and GO/Iron-oxide nanocomposites.

**Figure 10:**
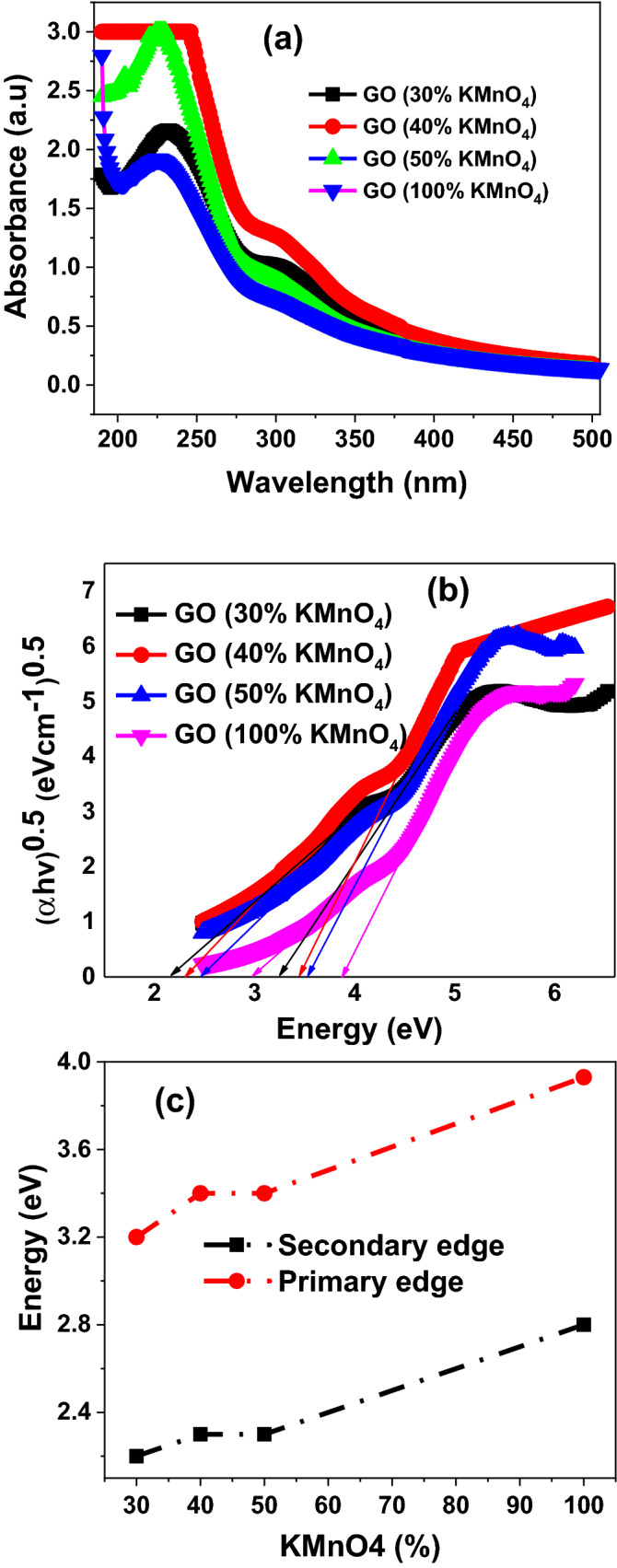
(**a**) Absorption spectra of controlled oxidized GO nanosheet samples (**b**) Tauc plots of controlled oxidized GO nanosheet samples (**c**) Variation in optical bandgap of controlled oxidized GO nanosheet samples versus amount of oxidizing agent.

Photoluminescence (PL) spectra is a well-known technique to study transfer process of the interface charge carrier as well as the recombination process involving the electron–hole pairs in semiconductor systems^59–61^ and PL emission results from the radiative recombination of excited electrons and holes. Room temperature (RT) PL spectrum of few selected controlled GO nanosheets samples with varying oxidizing agent is presented in Fig. 9a, while Fig. 9b showed RT PL spectrum of GO (i.e. 100% KMnO_4_), iron oxide nanoparticles and GO/Iron-oxide nanocomposites samples. We noted that the nanosheets based on GO systems showed strong PL intensity at wavelength of ~ 420 nm as reported earlier^62^. This high intensity represents the high recombination rate of photo generated charge carriers. While we observed that Iron oxide nanoparticles on the other hand do not show any PL intensity in this region. PL spectra of 95%GO-5% iron oxide and 93% GO-7% iron oxide nanocomposites represented in Fig. 9b gives us a great insight about understanding the electronic interaction among the iron oxide nanoparticles and GO flakes. Another interesting result we noticed that the PL intensity of the GO/Iron-oxide nanocomposites decreased with the increasing mass ratios of iron oxide nanoparticles, indicating that the recombination of photo generated electron–hole pairs is efficiently hindered. Our data from Fig. 10b reflects that 5% nanocomposite shows relatively lesser PL intensity as compared to the pure GO but for only 2% more loading as compared to 5% nanocomposite, 7% iron oxide-GO nanocomposite shows a drastic decrease in PL intensity, meaning the better separation of photo generated charge carriers.

### Optical absorption studies

**Figure 11:**
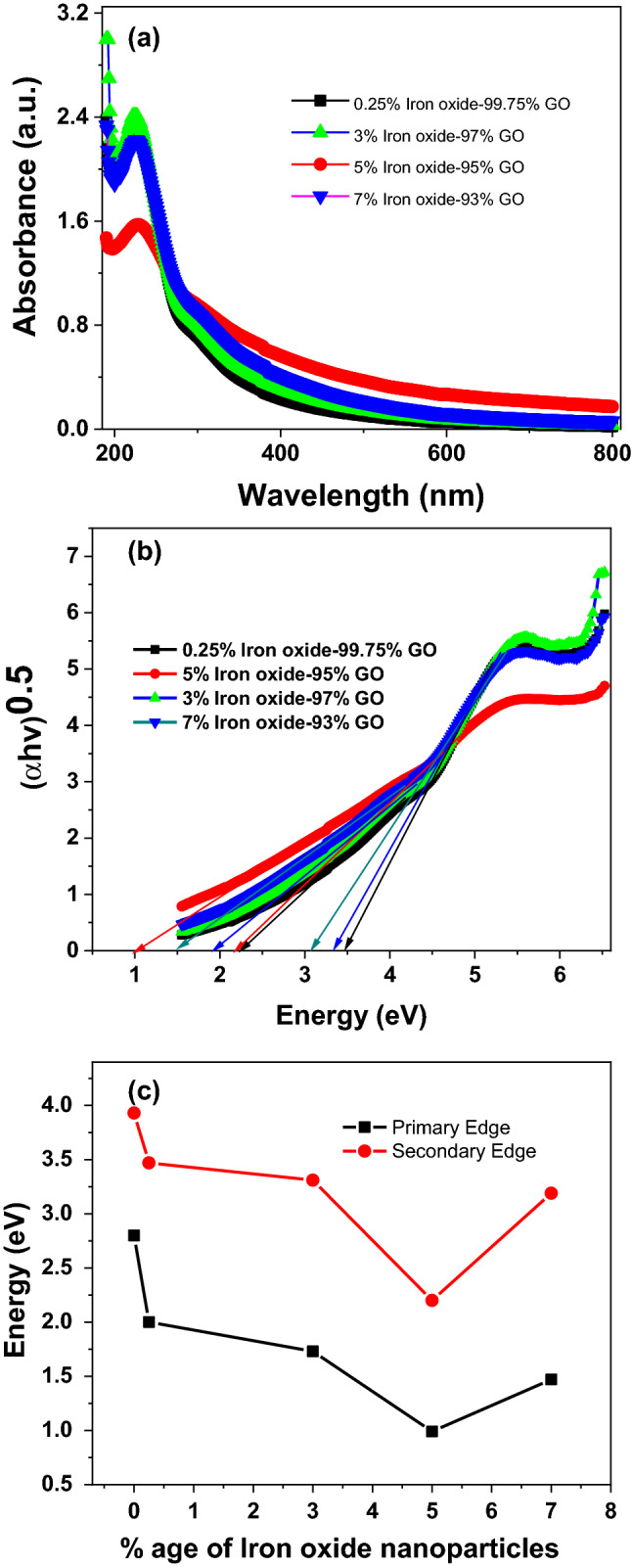
(**a**) Absorption spectra of composite samples (**b**) Tauc plot of nanocomposite samples (**c**) Band gap variations in nanocomposite samples with varying iron oxide content.

To identify the optical bandgap of synthesized GO nanosheets samples, UV–Visible absorption spectrophotometry at room temperature was carried out in the range of 190–700 nm. Figure 10a represented absorption spectra of a series of well controlled oxidized GO nanosheets samples for various concentrations of KMnO_4._ Similarly, we have obtained the UV–Visible absorption data for the series of GO/Iron-oxide nanocomposite samples as demonstrated in Fig. 11a. From absorption data we noted that overall more prominent and sharp absorption occurred around ~ 226 nm and this sharp peak represents the ordered graphitic structure with a $\uppi$ → $\uppi$* transition of aromatic C=C bonds present in the GO structure^63,64^. Another less intense absorption band occurred at ~ 300 nm that reported previously for n → $\uppi$* transition and commonly assigned to either C–O–C bonds or C=O bonds and therefore provides evidence for oxygen functionality on the GO sheets^63–65^. We were unable to measure the absorption spectra of GO (20% KMnO_4_) sample. The reduced amount of oxidizing agent may have induced the hydrophobic behavior in GO nanosheets that may decrease *sp*^2^/*sp*^3^ ratio in this sample, which eliminates the ability of this sample to get dispersed in deionized water. In all the specimens of controlled oxidized GO samples no absorption peak is observed in the range of 400–500 nm. Existence of absorption peaks in this region corresponds to KMnO_4_^62^.

To calculate the optical bandgap from this absorption data linear extrapolation of Tauc plot is used^66^ as;

5 $$\alpha hv={A(hv-{E}_{g})}^{n}$$

where $\mathrm{\alpha }$ the absorption coefficient, h is the frequency of light, A is proportionality constant, ${\mathrm{E}}_{\mathrm{g}}$ is the band-gap energy, and n is the type of electron excitation. Its value is different for direct and indirect band transition. Usually, “n” is 1/2 for direct bandgap and 2 for indirect bandgap transitions^67,68^. In our case we are only concerned with direct band to band transition of electron. Tauc plot of each controlled oxidized GO samples and GO/Iron-oxide nanocomposite samples have been extracted from absorption data and revealed in Figs. 10b and 11b respectively. We found and measured two distinct bandgap for each GO sample as reported earlier^63,69^. We denoted these bandgap with names primary edge (higher values of bandgap) and secondary edge (lower values of bandgap) as presented in Figs. 10b and 11b respectively for controlled oxidized GO samples and GO/Iron-oxide nanocomposite samples.

We noticed that both edges of each sample are increasing with increasing the concentration of KMnO_4_ as displayed in Fig. 10c. It was found that owing to the crude nature of the synthesis of GO nanosheets degree of oxidation of no two flakes is identical. One flake gets completely oxidized while the other still have an un-oxidized *sp*^2^ graphitic domain in it. This uneven *sp*^2^ to *sp*^3^ ratio may be the reasons behind two distinct bandgap of GO nanosheets^63,70,71^. In other words, by decreasing concentration of oxidizing agent, there is shrinkage in both band edges of each GO nanosheet samples that may be due to altering the *sp*^2^/*sp*^3^ ratio or may be the lesser amount of functional groups attached to the basal plane of GO nanosheets^42^.At this point if the amount of oxidant is further decreased exfoliation efficiency got effected and the graphite flacks gets only edge oxidation^24^. This rise in *sp*^2^ domain size induces hydrophobic behavior in to the material resulting in the loss of ability of the synthesized GO to dissolve in the polar solvents under normal conditions. Due to this reason UV absorption spectra could not be found for the samples made using less than 30% (1.8 wt. equiv.) of KMnO_4_. Here, we found an interesting correlation and trend that interplanar distance (extracted from XRD data), FWHM of D-band/G-band (dig out from Raman spectra) and band edges (calculated from UV–Vis absorption data) are increased with increasing the concentration of oxidizing agent KMnO_4_ and vice versa.

Figure 11c represents the relative band gaps of GO nanosheets and GO/Iron-oxide nanocomposite samples. Sudden decrease in bandgap values of 0.25% iron oxide-99.75%GO nanocomposite is observed for both the secondary and primary band edges from 3.93 and 2.8 eV of pure GO to 3.47 and 2.2 eV respectively. This sudden change in electronic property even for 0.25% loading of iron oxide nanoparticles is because of the greater surface area of nanoparticles that allows greater interaction and more possibility of electronic transfer. Same trend was observed for other nanocomposite samples. Tauc plot of as synthesized Iron Oxide nanoparticles were plotted (not shown here) and the linear extrapolation of this graph gives a value of 1.5 eV which is smaller than 1.92 eV of Fe_2_O_3_^72^ and 2.18 eV of Fe_3_O_4_^67^. Presence of both the $\mathrm{\alpha }$-Fe_2_O_3_ and Fe_3_O_4_ phases in specimen indicated by the XRD results or the greater crystallite size of these particles also calculated with the help of XRD could be a reason behind this decreased band gap value. This decrease in optical band gap is linear as the loading of iron nanoparticles is increased. But for 5% iron oxide/95% GO nanocomposite we have observed a huge decrease in both the major and minor bandgaps. This decrease in bandgaps is even greater than that of 7% iron oxide/93% GO nanocomposites. XRD analyses of 5% iron oxide-95% GO eliminates the possibility of bandgap reduction because of the increased *sp*^2^/*sp*^3^ ratio caused by the in situ reduction of GO flakes during nanocomposite synthesis process. Bandgaps of 7% nanocomposite is greater than the bandgap of 5% nanocomposite. This sudden increase in bandgap apart from increased loading reveals that 5% loading of iron oxide nanoparticles in GO is an optimum loading amount at which the nanocomposite shows the most decrease in bandgap values. These results have been verified by repeating the sample synthesis and characterizations at same parameters. XRD analysis performed previously reveals that GO is not reduced into r-GO in our composite sample formation. So the only reason behind any change in optical bandgap is because of the electronic transitions between the Fe_2_O_3_/Fe_3_O_4_ nanoparticles and GO nanosheets in nanocomposite samples.

**Figure 12:**
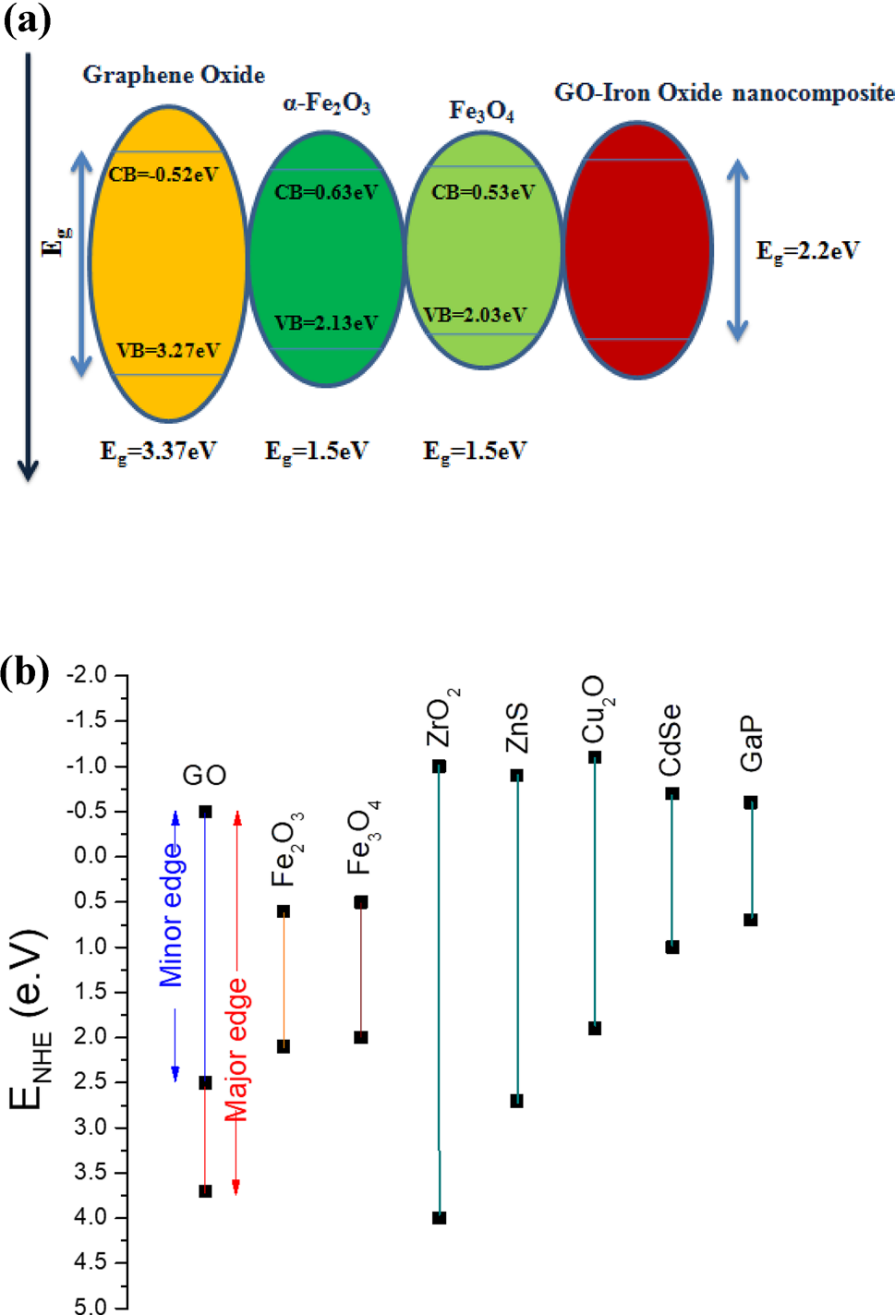
(**a**) Schematic representation of band edges (**b**) Band edge positions on Neutral hydrogen scale.

The optical bandgap of GO is far greater than it is required to operate as a semiconductor in optoelectronic devices. This bandgap can be reduced by controlling oxidation level in GO but it has its limitations as discussed. Another way to reduce is optical bandgap is by the incorporation of nano-scaled oxide particles that have a narrow energy gap^73^. Iron Oxide nanoparticles have ability to reduce GO in r-GO. In this article, we have carefully devised a method to incorporate Iron oxide nanoparticles in GO without its in-situ reduction. Mechanism behind this reduction in bandgap can be deeply understood by understanding band edge positions of iron oxide nanoparticles and GO. Band edge position on NHE are theoretically calculated and are shown in Fig. 12 and given in Table 1. Conduction band edge and valence band edge potentials for α-Fe_2_O_3_ and Fe_3_O_4_ are calculated using the absorption data and using the equation given below^74^

6 $${\text{E}}_{{{\text{cb}}}} \left( {{\text{Fe}}_{{2}} {\text{O}}_{{3}} } \right) = \chi \left( {{\text{Fe}}_{{2}} {\text{O}}_{{3}} } \right){-}{\text{E}}^{{\text{c}}} {-}0.{\text{5 E}}_{{\text{g}}}$$

7 $${\text{E}}_{{{\text{cb}}}} \left( {{\text{Fe}}_{{3}} {\text{O}}_{{4}} } \right) = \chi \left( {{\text{Fe}}_{{3}} {\text{O}}_{{4}} } \right){-}{\text{E}}^{{\text{c}}} {-}0.{\text{5 E}}_{{\text{g}}}$$

8 $${\text{E}}_{{{\text{vb}}}} = {\text{E}}_{{\text{g}}} {-}{\text{E}}_{{{\text{cb}}}} \left( {{\text{Fe}}_{{3}} {\text{O}}_{{4}} } \right)$$

9 $${\text{E}}_{{{\text{vb}}}} = {\text{E}}_{{\text{g}}} {-}{\text{E}}_{{{\text{cb}}}} \left( {{\text{Fe}}_{{2}} {\text{O}}_{{3}} } \right)$$

where χ represents absolute electronegativity of the corresponding material. χ for α-Fe_2_O_3_ is 5.88 eV and for Fe_3_O_4_ value of χ is 5.78 eV^75^. E^c^ is the scaling factor for converting absolute vacuum scale to NHE and its value is 4.5 eV. Band edge position of GO are taken from ^76^. Band edge positions given in Fig. 12 provide us insight about the mechanism governing the decrease in bandgap of the nanocomposite. The conduction band edge of GO mainly formed by the anti-bonding π* orbitals is at − 0.52 eV, whereas for α-Fe_2_O_3_ and Fe_3_O_4_ lies significantly lower at 0.63 and 0.53 eV respectively. Because of the variations in stoichiometry and small defects of Oxygen atoms α-Fe_2_O_3_ and Fe_3_O_4_ shows n-type behavior^77^. These thermally majority career electrons present in conduction band of iron oxide nanoparticles when interacts with the incident photons jumps to the conduction band of GO. Due to this lower band edge value, transfer of electrons in between different bands in nanocomposite is feasible. This transfer of electrons might be the reason behind the resultant reduced bandgap of GO-iron oxide nanocomposite as the difference in energy of these potentials is similar to the observed bandgap.

**Table 1:** Band edge positions of Graphene oxide, Fe_3_O_4_ and α-Fe_2_O_3_.

	GO major edge	GO minor edge	$\alpha -$Fe_2_O_3_	Fe_3_O_4_
Conduction band potential (eV)	− 0.52	− 0.52	0.63	0.53
Valence band potential (eV)	3.27	1.98	2.13	2.03
Band gap energy (eV)	3.37	2.54	1.5	1.5

## Conclusion

In this report, we conclude that it is possible to alter the optical bandgap of GO without subjecting it to thermal or chemical reduction. To tailor optical bandgap of GO, four samples of GO are synthesized by Improved Hummer's method utilizing 100, 40, 30 and 20% of the prescribed amount of KMnO_4_. X-ray diffraction (XRD) examination discloses the presence small un-oxidized graphitic domain (002) with prominent (001) plane of GO all over the flakes which in turn affects the exfoliation efficiency and hydrophilic properties except at 100% KMnO_4_ we observed pure GO (001) nanosheets. The XRD analysis also proves this controlled oxidation as the interplanar distance is found to decrease along with the oxidizing agent. But the presence of (002) Graphite peak in lower oxidized GO nanosheets points towards the shortcoming of this process. Raman spectroscopy reveals a linear decrease in I_D_/I_G_ ratio and linear increase in FWHM of D and G peaks as the amount of KMnO_4_ is increased, representing a linear increase in degree of oxidation or *sp*^2^/*sp*^3^ hybridization ratio. Optical bandgap calculated with the help of Absorption spectroscopy also shows a linear decrease as the amount of KMnO_4_is decreased. Limited control over the optical bandgap and compromised exfoliation efficiency made us to look for alternative approaches such as nanocomposites of GO with iron oxide nanoparticles.

A novel synthesis process for GO/Iron-oxide nanocomposites named as “Wet impregnation method” is devised that uses pre-synthesized Iron oxide nanoparticles. XRD analysis of synthesized nanocomposites ruled out the possibility of in situ reduction of GO. SEM ruled out all the possibilities of agglomeration of iron oxide nanoparticles and coagulation of GO nanosheets. EDX analysis confirmed the wt.% of constituent elements in all samples while elemental mapping showed the even distribution of nanoparticles throughout the GO nanosheets. Raman spectroscopy confirmed the fairly constant I_D_/I_G_ ratio and FWHM of D and G peaks, thus proving the fact that the synthesis process of nanocomposites has no effect on the degree of oxidation of GO flakes. Red shift in G peak position of all the nanocomposites synthesized using Wet impregnation method showed the electronic interaction among the constituents of the nanocomposite. Linear decrease in the intensity of PL spectra as the loading of Iron oxide nanoparticles is increased points toward the increased interaction among the iron oxide nanoparticles and GO flakes. Optical bandgap revealed the linear decrease with increasing of Iron oxide nanoparticles from 0.25 to 7% and we noted maximum shift to 0.99 eV in primary bandgap edge of 5% iron-oxide/95% GO nanosheet sample.

## Methods

### Synthesis of graphene oxide (GO)

According to Ayrat et al.^24^ Potassium Permanganate(KMnO_4_) is the main oxidizing agent in the synthesis of GO by Tour’s method (Improved Hummer’s method) and by changing the amount of KMnO_4_ degree of oxidation could be altered. We used improved hummer's method but instead of using complete 6 wt. equiv. of KMnO_4_ decreased amount is used. Four experiments were carried out by incorporating 3 (50%-GO), 2.4 (40%-GO), 1.8 (30%-GO) and 1.2 (20%-GO)wt. equiv. of KMnO_4_ or in other words 1.5, 1.2, 0.9 and 0.6 g of KMnO_4_ was used for each 0.5 g batch of graphite. Apart from this modification, each step was followed and every composition was followed according to the prescribed Tour’s or Improved hummer’s method.

To synthesize GO nanosheets, 2-g of graphite powder was slowly added in to the acid mixture of 270-ml of concentrated sulfuric acid (H_2_SO_4_) and 30-ml of concentrated phosphoric acid (H_3_PO_4_). During that addition vigorous stirring is done with the help of magnetic stirrer to avoid agglomeration. In this step acid molecules seep between the graphite layers and thus confirmed making an intercalation compound. After that the solution was kept in an ice bath to lower its temperature to about 10 °C. As the temperature drops 12-g of Potassium Permanganate (KMnO_4_) was slowly added in to the acid solution in such a manner that temperature never rises above 20 °C during this exothermic reaction. After this addition, solution turned to dark green color. To achieve complete oxidation, solution was heated to about 50 °C and stirred for 12 h. Then 250 ml of deionized water was slowly added while keeping the mixture on ice bath followed by the addition of 6 ml hydrogen peroxide (H_2_O_2_). This H_2_O_2_ neutralizes the oxidizing permanganate ions and stops the oxidation reaction, which was indicated by the change in color from dark green to yellow. At this point solution was washed with deionized water with the help of a centrifuge until the pH of the solution reaches to 7. The resultant slurry is then dried in a vacuum oven below 60 °C. Dried GO powders were finely ground and used for further characterizations.

### Synthesis of iron oxide nanoparticles

To synthesize iron oxide nanoparticles co-precipitation technique was used^78^. In this regard aqueous solutions of FeCl_3_ 6H_2_O (1.54 g/10 ml) and FeCl_2_ (0.56 g/10 ml) were made and added in to 150 ml of deionized water to start precipitation 25% ammonium hydroxide (NH_4_OH) was added slowly and pH of solution was maintained to 10. Solution was heated to 90 °C and 3-ml Hydrazine hydrate was added to prevent further oxidation of Fe_3_O_4_ nanoparticles and stirred for four hours then products was cooled to room temperature and filters washed with the help of repeated deionized water and absolute alcohol cycles. The remaining black brownish precipitates were then annealed at 450 °C for 3 h to improve crystallinity.

### Synthesis of GO/Iron-oxide nanocomposites

For the synthesis of nanocomposites series of GO/Iron-oxide, the suspensions of GO and Iron-oxide nanoparticles were mixed together in DI water with the help of ultra-sonicator bath in a ratio defined by the given empirical formula;

10 $${\text{GO}}_{{\left( {1 - {\text{x}}} \right)}} - {\text{Fe}}_{2} {\text{O}}_{3} ._{{\left( {\text{x}} \right)}}$$

This sonication was performed at room temperature for 45 min. The resultant was then placed in autoclave at 65 °C for two hours and then dried in a hot air oven at 100 °C.

### Characterization techniques

In this study Powder X-ray diffractometer from BRUKER D8 was used to perform the measurements. This diffractometer have a Copper (Cu) X-ray tube and an incident beam graphite monochromator, with Cu K(α) radiation operating at 45 kV and 40 mA. All the samples were scanned for 2θ value of 5$^\circ$ to 80$^\circ$ with a scanning resolution of 0.02$^\circ$.UV–vis absorption spectra (1 nm resolution) were acquired using UV–vis spectroscopy (Analytik Jena Specord 200 Plus UV–vis–NIR absorption spectrometer), utilizing quartz cuvettes with optical path lengths of 10 mm. For analysis 70 ppm solutions of all the materials are made using deionized water as a solvent. These solutions were then subjected to 25 min of ultra-sonication to achieve complete dispersion of the material. This process is carried out at room temperature to avoid thermally induced reduction of GO. Field Emission Scanning Electron Microscopy (FE-SEM) and energy dispersive spectroscopy (EDS) measurements were performed to study morphology and elemental distribution of GO and GO/Iron oxide nanocomposite. SEM was performed at a resolution of 10um, 5um, 2um and 1um.Raman spectrophotometer was used to observe degree of oxidation in our samples at room temperature with LASER excitation wavelength of 514 nm.

## Supplementary Information

Supplementary Information.

## Data Availability

The datasets used and/or analyzed during the current study available from the corresponding author on reasonable request.

## References

[1] Jung I, Field DA, Clark NJ, Zhu Y, Yang D, Piner RD, Stankovich S, Dikin DA, Geisler H, Ventrice CA, Ruoff RS (2009). Reduction kinetics of graphene oxide determined by electrical transport measurements and temperature programmed desorption. J. Phys. Chem. C.

[2] Boukhvalov DW, Katsnelson MI (2008). Modeling of graphite oxide. J. Am. Chem. Soc..

[3] Ito J, Nakamura J, Natori A (2008). Semiconducting nature of the oxygen-adsorbed graphene sheet. J. Appl. Phys..

[4] Luo Z, Vora PM, Mele EJ, Johnson AC, Kikkawa JM (2009). Photoluminescence and band gap modulation in graphene oxide. Appl. Phys. Lett..

[5] Hunt A, Dikin DA, Kurmaev EZ, Lee YH, Luan NV, Chang GS, Moewes A (2014). Modulation of the band gap of graphene oxide: The role of AA-stacking. Carbon.

[6] Wang C, Huang M, Ruoff RS (2021). Graphene oxide aerogel ‘ink’ at room temperature, and ordered structures by freeze casting. Carbon.

[7] Wang C, Chen X, Wang B, Huang M, Wang B, Jiang Y, Ruoff RS (2018). Freeze-casting produces a graphene oxide aerogel with a radial and centrosymmetric structure. ACS Nano.

[8] Jiang Y, Guo F, Liu Y, Xu Z, Gao C (2021). Three-dimensional printing of graphene-based materials for energy storage and conversion. SusMat.

[9] Chen X, Li W, Luo D, Huang M, Wu X, Huang Y, Lee SH, Chen X, Ruoff RS (2017). Controlling the thickness of thermally expanded films of graphene oxide. ACS Nano.

[10] Li K, Hu Z, Zhao R, Zhou J, Jing C, Sun Q, Rao J, Yao K, Dong B, Liu X, Li H (2021). A multidimensional rational design of nickel–iron sulfide and carbon nanotubes on diatomite via synergistic modulation strategy for supercapacitors. J. Colloid Interface Sci..

[11] He H, Ma K, Liu H, Li J, Zheng L, Zhang F, Fan X, Peng W, Ji J, Li Y (2022). Unraveling the amine oxidative coupling activity of hierarchical porous Fe–N_4_–O_1_ single-atom catalysts: oxygen atom-mediated dual reaction pathway. J. Mater. Chem. A.

[12] Peng X, Zhang T, Zheng J, Lv X, Zhang H, Hu JQ, Tian W, Tan S, Ji J (2021). Centrifugal force regularized laponite@ graphene hybrid membranes with ordered interlayer mass transfer channels and high structural stability for high-rate supercapacitors. Ind. Eng. Chem. Res..

[13] Zhang T, Liu P, Zhong Y, Zheng J, Deng K, Lv X, Li H, Tian W, Ji J (2022). N, S co-doped branched carbon nanotubes with hierarchical porous structure and electron/ion transfer pathways for supercapacitors and lithium-ion batteries. Carbon.

[14] Lv XB, Xie R, Ji JY, Liu Z, Wen XY, Liu LY, Hu JQ, Ju XJ, Wang W, Chu LY (2020). A novel strategy to fabricate cation-cross-linked graphene oxide membrane with high aqueous stability and high separation performance. ACS Appl. Mater. Interfaces.

[15] Xie J, Zhang J, Li S, Grote F, Zhang X, Zhang H, Wang R, Lei Y, Pan B, Xie Y (2013). Controllable disorder engineering in oxygen-incorporated MoS_2_ ultrathin nanosheets for efficient hydrogen evolution. J. Am. Chem. Soc.

[16] Liu S, Li F, Li Y, Hao Y, Wang X, Li B, Liu R (2017). Fabrication of ternary g-C_3_N_4_/Al_2_O_3_/ZnO heterojunctions based on cascade electron transfer toward molecular oxygen activation. Appl. Catal. B Environ..

[17] Fu L, Xiao X, Wang A (2018). Reduced graphene oxide coupled with g-C_3_N_4_ nanodots as 2D/0D nanocomposites for enhanced photocatalytic activity. J. Phys. Chem. Solids.

[18] Merazga A, Al-Zahrani J, Al-Baradi A, Omer B, Badawi A, Al-Omairy S (2020). Optical band-gap of reduced graphene oxide/TiO_2_ composite and performance of associated dye-sensitized solar cells. Mater. Sci .Eng. B.

[19] Hasan MT, Senger BJ, Ryan C, Culp M, Gonzalez-Rodriguez R, Coffer JL, Naumov AV (2017). Naumov optical band gap alteration of graphene oxide via ozone treatment. Sci. Rep..

[20] Velasco-Soto MA, Pérez-García SA, Alvarez-Quintana J, Cao Y, Nyborg L, Licea-Jiménez L (2015). Selective band gap manipulation of graphene oxide by its reduction with mild reagents. Carbon.

[21] Timoumi A (2018). Reduction band gap energy of TiO_2_ assembled with graphene oxide nanosheets. Graphene.

[22] Zhang J, Song H, Zeng D, Wang H, Qin Z, Xu K, Pang A, Xie C (2016). Facile synthesis of diverse graphene nanomeshes based on simultaneous regulation of pore size and surface structure. Sci. Rep..

[23] Marcano DC, Kosynkin DV, Berlin JM, Sinitskii A, Sun Z, Slesarev A, Alemany LB, Lu W, Tour JM (2010). Improved synthesis of graphene oxide. ACS Nano.

[24] Dimiev AMT, James M (2014). Mechanism of graphene oxide formation. ACS Nano.

[25] Cullity BD (1978). Elements of X-Ray Diffraction.

[26] Takagi H, Maruyama K, Yoshizawa N, Yamada Y, Sato Y (2004). XRD analysis of carbon stacking structure in coal during heat treatment. Fuel.

[27] Manoj B, Kunjomana AG (2012). Study of stacking structure of amorphous carbon by X-ray diffraction technique. Int. J. Electrochem. Sci.

[28] Feret FR (1998). Determination of the crystallinity of calcined and graphitic cokes by X-ray diffraction. Analyst.

[29] Kun P, Wéber F, Balázsi C (2011). Preparation and examination of multilayer graphene nanosheets by exfoliation of graphite in high efficient attritor mill. Central Eur. J. Chem..

[30] Sharma VK, Chattopadhyay MK, Kumar R, Ganguli T, Tiwari P, Roy SB (2007). Magnetocaloric effect in Heusler alloys Ni_50_Mn_34_In_16_ and Ni_50_Mn_34_Sn_16_. J. Phys. Condens. Matter.

[31] Jalili R, Esrafilzadeh D, Aboutalebi SH, Sabri YM, Kandjani AE, Bhargava SK, Della Gaspera E, Gengenbach TR, Walker A, Chao Y, Wang C (2018). Silicon as a ubiquitous contaminant in graphene derivatives with significant impact on device performance. Nat. Commun..

[32] Venugopal G, Jung MH, Suemitsu M, Kim SJ (2011). Fabrication of nanoscale three-dimensional graphite stacked-junctions by focused-ion-beam and observation of anomalous transport characteristics. Carbon.

[33] Wang YY, Ni ZH, Yu T, Shen ZX, Wang HM, Wu YH, Chen W, Shen Wee AT (2008). Raman studies of monolayer graphene: the substrate effect. J. Phys. Chem. C.

[34] Pimenta MA, Dresselhaus G, Dresselhaus MS, Cancado LG, Jorio A, Saito R (2007). Studying disorder in graphite-based systems by Raman spectroscopy. Phys. Chem. Chem. Phys..

[35] Xue Y, Wu B, Jiang L, Guo Y, Huang L, Chen J, Tan J, Geng D, Luo B, Hu W, Yu G (2012). Low temperature growth of highly nitrogen-doped single crystal graphene arrays by chemical vapor deposition. J. Am. Chem. Soc..

[36] Eda G, Fanchini G, Chhowalla M (2008). Large-area ultrathin films of reduced graphene oxide as a transparent and flexible electronic material. Nat. Nanotechnol..

[37] Bai S, Chen S, Shen X, Zhu G, Wang G (2012). Nanocomposites of hematite (α-Fe_2_O_3_) nanospindles with crumpled reduced graphene oxide nanosheets as high-performance anode material for lithium-ion batteries. RSC Adv..

[38] Chaunchaiyakul S, Yano T, Khoklang K, Krukowski P, Akai-Kasaya M, Saito A, Kuwahara Y (2016). Nanoscale analysis of multiwalled carbon nanotube by tip-enhanced Raman spectroscopy. Carbon.

[39] Tuinstra F, Koenig JL (1970). Raman spectrum of graphite. J. Chem. Phys..

[40] Dey A, Athar J, Varma P, Prasant H, Sikder AK, Chattopadhyay S (2015). Graphene-iron oxide nanocomposite (GINC): An efficient catalyst for ammonium perchlorate (AP) decomposition and burn rate enhancer for AP based composite propellant. RSC Adv..

[41] Ferrari AC, Robertson J (2000). Interpretation of Raman spectra of disordered and amorphous carbon. Phys. Rev. B.

[42] Krishnamoorthy K, Veerapandian M, Yun K, Kim SJ (2013). The chemical and structural analysis of graphene oxide with different degrees of oxidation. Carbon.

[43] Wan YH, Shi XQ, Xia H, Xie J (2013). Synthesis and characterization of carbon-coated Fe_3_O_4_ nanoflakes as anode material for lithium-ion batteries. Mater. Res. Bull..

[44] Wang WZ, Wang GH, Wang XS, Zhan YJ, Liu YK, Zheng CL (2002). Synthesis and characterization of Cu_2_O nanowires by a novel reduction route. Adv. Mater..

[45] Serrano A, Fernández JF, de la Fuente OR, García MA (2017). Materials today chemistry. Mater. Today.

[46] Fan HM, You GJ, Li Y, Zheng Z, Tan HR, Shen ZX, Tang SH, Feng YP (2009). Shape-controlled synthesis of single-crystalline Fe_2_O_3_ hollow nanocrystals and their tunable optical properties. J. Phys. Chem. C.

[47] Zhou G (2012). Oxygen bridges between NiO nanosheets and graphene for improvement of lithium storage. ACS Nano.

[48] Zhou J, Song H, Ma L, Chen X (2011). Magnetite/graphene nanosheet composites: interfacial interaction and its impact on the durable high-rate performance in lithium-ion batteries. RSC Adv..

[49] Niyogi S, Bekyarova E, Itkis ME, Zhang H, Shepperd K, Hicks J, Sprinkle M, Berger C, Lau CN, Deheer WA, Conrad EH (2010). Spectroscopy of covalently functionalized graphene. Nano Lett..

[50] Dresselhaus MS, Jorio A, Hofmann M, Dresselhaus G, Saito R (2010). Perspectives on carbon nanotubes and graphene Raman spectroscopy. Nano Lett..

[51] Sharma R, Alam F, Sharma AK, Dutta V, Dhawan SK (2014). ZnO anchored graphene hydrophobic nanocomposite-based bulk heterojunction solar cells showing enhanced short-circuit current. J. Mater. Chem. C.

[52] Yu H, Wang T, Wen B, Lu M, Xu Z, Zhu C, Chen Y, Xue X, Sun C, Cao M (2012). Graphene/polyaniline nanorod arrays: synthesis and excellent electromagnetic absorption properties. J. Mater. Chem..

[53] Liao Y, Zhang C, Wang X, Li XG, Ippolito SJ, Kalantar-Zadeh K, Kaner RB (2011). Carrier mobility of single-walled carbon nanotube-reinforced polyaniline nanofibers. J. Phys. Chem. C.

[54] Li H, Xu T, Wang C, Chen J, Zhou H, Liu H (2005). Tribochemical effects on the friction and wear behaviors of diamond-like carbon film under high relative humidity condition. Tribol. Lett..

[55] Adenier A, Bernard MC, Chehimi MM, Cabet-Deliry E, Desbat B, Fagebaume O, Pinson J, Podvorica F (2001). Covalent modification of iron surfaces by electrochemical reduction of aryldiazonium salts. J. Am. Chem. Soc..

[56] Krishnamoorthy K, Veerapandian M, Zhang LH, Yun K, Kim SJ (2012). Antibacterial efficiency of graphene nanosheets against pathogenic bacteria via lipid peroxidation. J. Phys. Chem. C.

[57] Knight DS, White WB (1989). Characterization of diamond films by Raman spectroscopy. J. Mater. Res..

[58] Cançado LG, Takai K, Enoki T, Endo M, Kim YA, Mizusaki H, Jorio A, Coelho LN, Magalhães-Paniago R, Pimenta MA (2006). General equation for the determination of the crystallite size L a of nanographite by Raman spectroscopy. Appl. Phys. Lett..

[59] Haldar KK, Sinha G, Lahtinen J, Patra A (2012). Hybrid colloidal Au-CdSe pentapod heterostructures synthesis and their photocatalytic properties. ACS Appl. Mater. Interfaces.

[60] Xing X, Liu R, Yu X, Zhang G, Cao H, Yao J, Ren B, Jiang Z, Zhao H (2013). Self-assembly of CdS quantum dots with polyoxometalate encapsulated gold nanoparticles: Enhanced photocatalytic activities. J. Mater. Chem. A.

[61] Shi Y, Li H, Wang L, Shen W, Chen H (2012). Novel α-Fe_2_O_3_/CdS cornlike nanorods with enhanced photocatalytic performance. ACS Appl. Mater. Interfaces.

[62] Teng C-Y, Yeh T-F, Lin K-I, Chen S-J, Yoshimura M, Teng H (2015). Synthesis of graphene oxide dots for excitation-wavelength independent photoluminescence at high quantum yields. J. Mater. Chem. C..

[63] Hsu HC, Shown I, Wei HY, Chang YC, Du HY, Lin YG, Tseng CA, Wang CH, Chen LC, Lin YC, Chen KH (2013). Graphene oxide as a promising photocatalyst for CO_2_ to methanol conversion. Nanoscale.

[64] Lai Q, Zhu S, Luo X, Zou M, Huang S (2012). Ultraviolet-visible spectroscopy of graphene Oxides. AIP Adv..

[65] Saxena S, Tyson TA, Shukla S, Negusse E, Chen H, Bai J (2011). Investigation of structural and electronic properties of graphene oxide. Appl. Phys. Lett..

[66] Parida KM, Nashim A, Mahanta SK (2011). Visible-light driven Gd_2_Ti_2_O_7_/GdCrO_3_ composite for hydrogen evolution. Dalton Trans..

[67] El Ghandoor H, Zidan HM, Khalil MM, Ismail MI (2012). Synthesis and some physical properties of magnetite (Fe_3_O_4_) nanoparticles. Int. J. Electrochem. Sci.

[68] Viezbicke BD, Patel S, Davis BE, Birnie DP (2015). Evaluation of the Tauc method for optical absorption edge determination: ZnO thin films as a model system. Phys. Status Solidi (b).

[69] Eda G, Chhowalla M (2010). Chemically derived graphene oxide: Towards large-area thin-film electronics and optoelectronics. Adv. Mater..

[70] Kumar P, Bansiwal A, Labhsetwar N, Jain SL (2015). Visible light assisted photocatalytic reduction of CO_2_ using a graphene oxide supported heteroleptic ruthenium complex. Green Chem..

[71] Kumar P, Sain B, Jain SL (2014). Photocatalytic reduction of carbon dioxide to methanol using a ruthenium trinuclear polyazine complex immobilized on graphene oxide under visible light irradiation. J. Mater. Chem. A.

[72] Misho RH, Murad WA (1992). Band gap measurements in thin films of hematite Fe_2_O_3_, pyrite FeS_2_ and troilite FeS prepared by chemical spray pyrolysis. Sol. Energy Mater. Sol. Cells.

[73] Yang X, Su X, Shen M, Zheng F, Xin Y, Zhang L, Hua M, Chen Y, Harris VG (2012). Enhancement of photocurrent in ferroelectric films via the incorporation of narrow bandgap nanoparticles. Adv. Mater..

[74] Xu Y, Schoonen MA (2000). The absolute energy positions of conduction and valence bands of selected semiconducting minerals. Am. Miner..

[75] Pearson RG (1988). Absolute electronegativity and hardness: Application to inorganic chemistry. Inorg. Chem..

[76] Kumar S, Kumar A, Bahuguna A, Sharma V, Krishnan V (2017). Two-dimensional carbon-based nanocomposites for photocatalytic energy generation and environmental remediation applications. Beilstein J. Nanotechnol..

[77] Medina-Ramírez I, Hernández-Ramírez A (2016). Photocatalytic Semiconductors.

[78] Sahu TK, Arora S, Banik A, Iyer PK, Qureshi M (2017). Efficient and rapid removal of environmental malignant arsenic (III) and industrial dyes using reusable, recoverable ternary iron oxide-ormosil-reduced graphene oxide composite. ACS Sustain. Chem. Eng..

