# Supplementary material for: Optimization of bandgap reduction in 2-dimensional GO nanosheets and nanocomposites of GO/iron-oxide for electronic device applications
Source: Sci Rep. 2023 Apr 28;13:6954. doi: 10.1038/s41598-023-33200-4 (PMC10147644; doi:10.1038/s41598-023-33200-4)
Supplement: Supplementary file 1 — Supplementary Information. [file 41598_2023_33200_MOESM1_ESM.docx]

## Supplementary Information

**Optimization of Bandgap Reduction in 2-Dimensional GO Nanosheets and Nanocomposites of GO/Iron-oxide for Electronic Device applications**

**Authors:** Sana Zainab^1†^, Muhammad Azeem^1^, Saif Ullah Awan^1*^, Syed Rizwan^2^, Naseem Iqbal^3^, Jamshaid Rashid^4, 5^

1. Department of Electrical Engineering, College of Electrical and Mechanical Engineering, National University of Sciences and Technology (NUST), Islamabad 44000, Pakistan.
2. Department of Physics, School of Natural Sciences (SNS), National University of Sciences and Technology (NUST), Islamabad 44000, Pakistan.
3. US-Pakistan Centre for Advanced Studies in Energy (USPCAS-E), National University of Sciences and Technology (NUST), Islamabad 44000, Pakistan.
4. Department of Environmental Sciences, Faculty of Biological Sciences, Quaid-i-Azam University, Islamabad 45320, Pakistan.
5. BNU-HKUST Laboratory for Green Innovation, Advanced Institute of Natural Sciences, Beijing Normal University at Zhuhai, Zhuhai 519087, China.

Corresponding Author: ^*^[saifullahawan@ceme.nust.edu.pk](mailto:saifullahawan@ceme.nust.edu.pk), [^*^ullahphy@gmail.com](mailto:*%20ullahphy@gmail.com), ^†^szanib.ee17ceme@student.nust.edu.pk

1. **Introduction**

Graphene oxide (GO) is a two dimensional (2D) wrinkled sheet of sp^3^ hybridized carbon atoms arranged in a hexagonal lattice with oxygen based functional groups attached to its basil plane. The presence of these functional groups reduces the interplanar forces and imparts hydrophilic character, thereby promoting complete exfoliation of single GO layers in aqueous and in some organic solvents ^1,2^. Nanosheets of GO is a promising alternative to Graphene. GO have a layered thickness of around 1 nm while the lateral dimension varying from a few nanometers to several micrometers. Low manufacturing cost, easy scale up potentials, its property to get easily dispersed in polar solvents and as a promising agent in forming composites had given great importance to GO not only for a source of Graphene but also as a material in its own right. Unlike Graphene, GO do not have *sp^2^* hybridized carbon atoms arranged in a honey comb lattice rather carbon atoms in GO have *sp^3^* hybridization and a large amount of functional groups such as hydroxyl, epoxy and carboxyl side groups attached to its surface. Owing to its production procedure GO also have a substantial amount of surface defects. To understand the structure of GO and to identify the functional groups attached to carbon atoms, contribution of Hofmann and Holst could not be neglected. In 1939 they proposed that only Epoxy group is present on the graphene surface^3^. But Ruess eliminates the possibility of a single type of functional group. He proposed a crystal model based on the experimental data that proved the presence of hydroxyl groups along with the epoxy groups^4^. This model also sheds light upon the distortion of *sp^3^*hybridization as the functional groups get attached to the carbon atom. These functional groups could attach on both sides of the GO sheets as forming molecular structure of GO. Depending upon the manufacturing method and the time of oxidation, the amount and type of functional groups can be controlled thus altering the electronic as well as mechanical properties of the GO^5^.

Suk et al. in 2010 for the first time determines the young modulus and pre-stress for free standing mono, bi and tri layers of GO using AFM imaging in contact mode. According to their analysis single layer GO have a Young’s modulus of about 156.5 GPa which is about one fifth of the Young’s modulus of pristine Graphene. Elastic stiffness of single layer is around 109 N/m using a thickness of 0.7 nm. The Young’s modulus of Bi and tri layered graphene is found to be about 223 GPa and 229 GPa respectively. This rise in Young’s modulus is strong indicator of strong inter planner interaction among the GO layers, which doesn’t allow the interlayer sliding. Mechanical properties of GO could be effected by the type of functional groups, degree of oxidation and molecular weight of the functional groups attached to the planes of honey comb lattice. Zheng et al. in 2010 used molecular mechanics and molecular dynamic simulation to analyze the elastic properties of GO. They found out that by increasing the amount of (-OH) hydroxyl groups in GO Young’s modulus decreases linearly. According to them strength of GO sheet is dependent on the *sp^2^/sp^3^* hybridization ratio. If the degree of oxidation is high it means that more functional groups are attached to the carbon atom by destroying the local Π bonds in the sheet. This bond breakage changes the hybridization from *sp^3^* to *sp^2^* effecting the Young’s modulus to decrease.

Under ambient conditions Hematite (α-Fe_2_O_3_) behaves as an indirect bandgap n-type semiconductor. Its bandgap is considered to be 2.3 eV but this value could be lower if the size of the nanoparticles increases above 20 nm. This happens because of the quantum confinement as the size decreases owing to the quantum confinement energy levels gets discretized and attains higher energy states thus increasing the bandgap of the material. On the other hand increased particle size in nanoparticles doesn’t let the energy levels to attain higher levels thus reducing the bandgap. Conduction band of Hematite consists of only the empty d-orbitals of Fe^3+^ and its valance band is made by the mixture of 3d filled orbitals of Fe^3+^ and 2p anti-bonding orbitals of Oxygen atom^6^. Magnetite (Fe_3_O_4_) is a naturally occurring mineral with a face centered cubic crystal structure. This black color n-type semiconductor unlike other iron oxides has iron in both Fe^3+^ and Fe^2+^ states. In the crystal of Magnetite half of the octahedral sites are occupied by the Fe^2+^ ions while the remaining half of these octahedral sites and some of the tetrahedral sites are occupied by the Fe^3+^ ions. In this mineral Fe^3+^ ions are about double in concentration as compared to Fe^2+^ ions. As these Fe^2+^ ions are replicable by other elements with a similar divalent ion, Fe_3_O_4_ could be doped as a p-type semiconductor.

Composites are materials that are made by the mixing of two or more different materials to combine the properties of the precursors. In some cases properties of composites out run the precursor’s properties. If any of the component or precursor used to make composites have a dimension which is in nanometer scale then the resultant is named as nanocomposite. In conventional composites we need about 40% loading to achieve some improvement in properties. Apart from conventional composites in nanocomposites we can have a change in properties for as low as 5% loading of the nanoparticles. This is because of the nano-scale dimensions of the particles, which give rise to quantum confinement. This phenomenon along with increased surface area which give rise to greater reactivity, are some of the causes for these improved properties of the nanocomposites. Classical theories don’t explain this phenomenon. Nanocomposites have a very strange type of structure, they could be thought as a solid material which has a phase dimensional repetition in a nanometer scale. Host material in a nanocomposite could be organic or inorganic and there is also no limitation for the component material. Both of these materials could exist in one or more phases. Only thing that matters is the nanometer scale dimension in either one, two or in three dimensions of the component material. It is quite possible that nanocomposites have properties such as physical, electrical, photocatalytic, electrochemical and optical that are totally different form their parent materials.

**2. X-ray Diffraction Analysis**

.

**Figure-1S:** XRD spectra of **(a)** commercial grade graphite**(b)** pure graphene oxide (GO) and **(c)** Iron oxide

**Acknowledgments**

The authors acknowledge the financial support from the Higher Education Commission of Pakistan under grant No: 5339/Federal/NRPU/R&D/HEC/2015 and project titled "Controlled Synthesis Of Two-Dimensional Nanosheets and Multilayer For Electronic Devices"

**Data availability statement:**

The datasets used and/or analysed during the current study available from the corresponding author on reasonable request

**Author Contribution**

S. Zainab and M. Azeem carried out the experimentation and analyzed the data, N. Iqbal and S. Rizwan helped in characterizations. J. Rashid helped in synthesis. S. Awan conceived the idea and supervised the research project

**Competing interest**

Authors declare no Competing Financial or Non-Financial Interests

**References**

1 Jalili, R. A., Seyed Hamed Esrafilzadeh, Dorna Konstantinov, Konstantin Moulton, Simon E Razal, Joselito M Wallace, Gordon G. Organic solvent-based graphene oxide liquid crystals: a facile route toward the next generation of self-assembled layer-by-layer multifunctional 3D architectures. *ACS Nano* 7, 3981-3990 (2013).

2 Vickers, N. J. Animal communication: when i’m calling you, will you answer too? *Current biology* 27, 713-715 (2017).

3 Hofmann, U. & Holst, R. Über die Säurenatur und die Methylierung von Graphitoxyd. *Berichte der deutschen chemischen Gesellschaft (A and B Series)* 72, 754-771 (1939).

4 Ruess, G. Über das graphitoxyhydroxyd (graphitoxyd). *Monatshefte für Chemie und verwandte Teile anderer Wissenschaften* 76, 381-417 (1947).

5 Compton, O. C. N., SonBinh T. Graphene oxide, highly reduced graphene oxide, and graphene: versatile building blocks for carbon‐based materials. *Small* 6, 711-723 (2010).

6 Zhang Z Boxall, C. K. G. in Colloids in the Aquatic Environment 145-163 (Elsevier, 1993).
